# Supplementary material for: RNA-peptide interactions tune the ribozyme activity within coacervate microdroplet dispersions
Source: Nat Commun. 2025 Oct 1;16:8765. doi: 10.1038/s41467-025-63656-z (PMC12489098; doi:10.1038/s41467-025-63656-z)
Supplement: Supplementary file 1 — Supplementary Information [file 41467_2025_63656_MOESM1_ESM.pdf]

RNA-peptide interactions tune ribozyme activity within coacervate microdroplets

Basusree Ghosh[#a], Patrick M McCall[#a,b,c,d,e], Kristian Kyle Le Vay[f], Archishman Ghosh[a,b,g], Lars Hubatsch[a], David T. Gonzales[a], Jan Brugués[a,b,c,d], Hannes Mutschler[f]*, T-Y Dora Tang[a,d,g]*

[a] Max Planck Institute of Molecular Cell Biology and Genetics, Pfotenhauerstrasse 108, 01307 Dresden, Germany
[b] Max Planck Institute for the Physics of Complex Systems, Nöthnitzer Strasse 38, 01187 Dresden, Germany
[c] Center for Systems Biology Dresden, Pfotenhauerstrasse 108, 01307 Dresden, Germany
[d] Physics of Life, Cluster of Excellence, TU Dresden, 01603 Dresden, Germany
[e] Leibniz Institute of Polymer Research Dresden, Hohe Straße 6, 01069 Dresden, Germany
[f] Department of Chemistry and Chemical Biology, TU Dortmund University, Dortmund, Germany
[g] Department of Synthetic biology, University of Saarland, Campus Saarbrücken, 66123, Saarbrücken, Germany.

[#] These authors contributed equally

*Corresponding authors: dora.tang@uni-saarland.de, hannes.mutschler@tu-dortmund.de

Contents

1.0. Chemicals and materials
2.0. Generation and selection of short cationic peptide sequences
2.1. Sequence generator script
3.0. Coacervate characterisation
3.1. Turbidity, droplet formation and zeta potential measurements
3.2. Supplementary Information Note 1: Discussion of zeta potential data
3.3. Discussion of droplet size analysis
4.0. Characterisation of RNA and determination of ribozyme kinetics
4.1. Testing ribozyme function in buffer
4.2. Characterisation of ribozyme kinetics in after coacervate formation
4.2.1. Characterisation of ribozyme kinetics from the pellet
4.2.2. Characterisation of ribozyme kinetics from the dispersion
4.3. Characterisation of ribozyme kinetics in buffer
4.4. Ribozyme reaction in the supernatant
4.5. Determination of RNA fraction in the dilute phase vs the pellet
4.6. Supplementary Information Note 2: Confirmation of product generation in the dense phase with the Scenario B model
4.6.1. Overview
4.6.2. The Scenario B model
4.6.3. Determination of parameter values for the Scenario B model
4.6.3.1. Volumes V_I^* and V_{II}
4.6.3.2. Equilibrium partition coefficient P_{sub}^{eq}
4.6.3.3. Equilibrium partition coefficient P_{prod}^{eq}
4.6.3.4. Effective rate constant $k_{I,\mathrm{eff}} = k_I \times c_{HH_{min}}^I$
4.6.3.5. Constant characterising rate of molecule exchange between phases, k_{ex}
4.6.4. Determination of initial conditions for the Scenario B model
4.6.5. Conversion between integrated intensity on the gel and number of molecules, θ
4.7. Detection of substrate and product with the FRET-substrate using confocal microscopy
5.0. Determination of tie-lines and HH_{min} and peptide concentrations at phase coexistence
5.1. Overview
5.2. Determination of tie-lines
5.2.1. Dilute-phase HH_{min} and peptide concentration measurements
5.2.2. Determination of tie-line parameters
5.3. Determination of condensed-phase HH_{min} and peptide concentrations
5.3.1. Error analysis and propagation
5.4. Supplementary Information Note 3: Implications from comparative tie-line analysis
5.5. Supplementary Information Note 4: Invariance of phase coexistence and increase in droplet size along tie-lines
5.6. Quantitative phase imaging and analysis
5.6.1. Determination of refractive index increments and partial specific volumes
5.7. Calculation of partition coefficient
5.8. Calculation of the coacervate-phase volume fraction and polymer volume fraction in the coacervate phase
6.0. Fluorescence recovery after photobleaching: ribozyme diffusion inside droplet
7.0. RNase A footprinting assay
8.0. Supplementary Information Note 5: Determination of pH within the coacervate droplet
8.1. Motivation and model
8.2. Experimental determination of pH^I and pH^{tot}
8.3. Generation of simulated pH^{II} data via Monte Carlo
8.4. Self-consistency assessment of physical limits applied to pH-related calculations
9.0 Uncropped gels from data presented in the main text and supplementary information
10.0. Associated content and data availability
11.0. Supplementary References

1.0 Chemicals and materials

Table S1: Chemicals and reagents used in this study.

Name	Company
Tris base (CAS 77-86-1)	Sigma-Aldrich, USA
Ethylenediaminetetraacetic acid (EDTA) (CAS 60-00-4)	Sigma-Aldrich, USA
Magnesium Chloride Hexahydrate (CAS 7791-18-6)	Merck Millipore, DE
Urea (CAS 57-13-6)	Merck Millipore, DE
Hydrochloric acid (CAS 7647-01-0)	Merck Millipore, DE
Boric acid (CAS 10043-35-3)	Sigma-Aldrich, USA
Nuclease-Free Water (Cat # AM9937)	Thermo Fisher Scientific Inc., USA
Ammonium peroxydisulphate (CAS 7727-54-0)	Carl Roth GmbH & Co., DE
40% Acrylamide/Bis Solution (19:1) (ID 1610144)	Bio-rad, USA
Tetramethylethylenediamine (TEMED) (CAS 110-18-9)	VWR International GmbH, USA
Ribonuclease A (CAS 9001-99-4)	Carl Roth GmbH & Co., DE
2X RNA gel loading dye (Cat # R0641)	Thermo Fisher Scientific Inc, USA
sticky-Slide 18 Well (Cat # 81818)	Ibidi GmbH, DE
Sodium hexametaphosphate (CAS 68915-31-1)	Sigma-Aldrich, USA
BCA Protein Assay Kit (ID 23227)	Thermo Fisher Scientific Inc., USA
Gel electrophoresis cassettes (ID 3459903)	Bio-rad, USA
384 well, F-bottom, black and transparent (ID 781946)	Greiner Bio-One GmbH, Austria
RNase kit (Cat # AM2283)	Thermo Fisher Scientific Inc., USA

All RNAs (Table S2) were purchased as RNase-free HPLC-purified oligos from either Eurofins Ebersberg, Germany or Integrated DNA Technologies (IDT, USA). Aliquots were prepared in Nuclease-free water and stored at -80 ^{o}C until use.

Table S2: RNA oligonucleotide sequences

Name	5' tag	Sequence (5'-3')	3' tag	Length	MW (g/mol)
HH_{min}		GGGAGCUGAACUGAUGAGUCCGUGAG GACGAAAGGCACA		39	12733.8
Mut-HH		GGGAGCUGAACU**A**AUGAGUCCGUGAG GAC**A**AAAGGCACA		39	12702
FAM-HH	FAM	GGGAGCUGAACUGAUGAGUCCGUGAG GACGAAAGGCACA		39	13271.2
FAM-subs	FAM	UGCCUCUUCAGC		12	4245.7
FRET-subs	FAM	UGCCUCUUCAGC	Alexa532	12	5065.5

Point mutations are indicated in bold.

2.0. Generation and selection of short cationic peptide sequences

A probabilistic sequence generator script (shown in section 2.1 below) was used to generate six peptide sequences from 12 amino acids. At each point in the sequence, the probability of inclusion is 0.25 for each of R and K, 0.1 for each of I and L, 0.09 for V, and 0.03 for each of D, E, S, T, G, A and P. On average, these probabilities yield sequences with 50% positive charge (R and K), 29% long chain aliphatic amino acids (I, V and L) and 21% acidic, polar and hydrophobic amino acids (D, E, S, T, G, A and P). The individual probabilities for the different amino acids were chosen to increase the chances of coacervate formation between the peptides and RNA. Recent studies have shown that the presence of hydrophobic amino acids (G, A, L, I and V) along with positively charged amino acids (K and R) facilitate coacervate formation[1 2]. Particularly, long-chain hydrophobic amino acids (V, I and L) with increased aliphatic index were found to be more efficient in coacervate formation compared to short-chain hydrophobic amino acids[2]. The seven peptides listed in Table S3 were purchased in HPLC-purified form from BIOMATIK, Ontario, Canada. All peptides were dissolved in nuclease-free water and the concentration was calculated by measuring the difference in far UV absorbance using the Waddell method[3], Peptide (µg/mL) = 144*(A_{215}-A_{225}).

2.1. Sequence generator script

```
import numpy as np
a_dict = {'R':0,'K':1,'I':2,'V':3,'L':4,'D':5,'E':6,'P':7,'S':8,'A':9,'T':10,'G':11}
a_dict = {v: k for k, v in a_dict.items()}
for i in range(150):
num_seq = np.random.choice(12, 13, p=[0.25,0.25,0.1,0.09,0.1,0.03,0.03,0.03,0.03,0.03,0.03,0.03])
print (''.join([a_dict[num] for num in num_seq]))
```

Table S3: Peptide sequences

Name	Sequence	MW (Da)	Number of positive charges
P-1	RSKKTPRGKSRKK	1556.85	8
P-2	RLRVRKRRPTKRG	1679.03	8
P-3	KRRDVIKRKPLRK	1693.12	7
P-4	SRIRKKAKRRKIV	1639.04	8
P-5	LIRKTKRRIKREK	1725.16	7
P-6	RVTKRKRISIKSK	1599.99	7
P-7	RKRKRKRKRKRKR	1880.37	13

3.0. Coacervate characterisation

3.1. Turbidity, droplet formation and zeta potential measurements

HH_{min} and peptide concentrations were prepared in units of charge concentration for all experiments unless stated otherwise. 2X composition of working buffer was used to prepare the reaction mixture for experiments to result in 1X composition (10 mM Tris, 1mM $MgCl_2$ at pH 8.1). To find the correct ratio for the coacervate formation, the turbidity of the RNA-peptide solution was measured using

UV-Vis absorbance at 500 nm with a Tecan Spark 20M microplate reader. 500 μM HH_{min} (polymer concentration * 39) was mixed in 384 well plates (flat bottom) with increasing concentration of peptides (0 – 5mM) at final volume of 20 μL buffer. The turbidity I_{turb} of the solution was calculated as a percentage from absorbance measurements at 500 nm (A_{500}) and 950 nm (A_{950}) according to

$$I_{turb} = 100 - T = 100 - 10^{2-A} \tag{3.1}$$

where $T = 10^{2-A}$ is the percentage of transmitted light and the calibrated absorbance A is given by

$$A = A_{500} - A_{950}. \tag{3.2}$$

Turbidity data was plotted as a function of peptide charge concentration for each peptide using GraphPad Prism 9.4 (Figure S1A). Unless otherwise stated, a 1:2 ratio of the HH_{min}: peptide was chosen for further experiments.

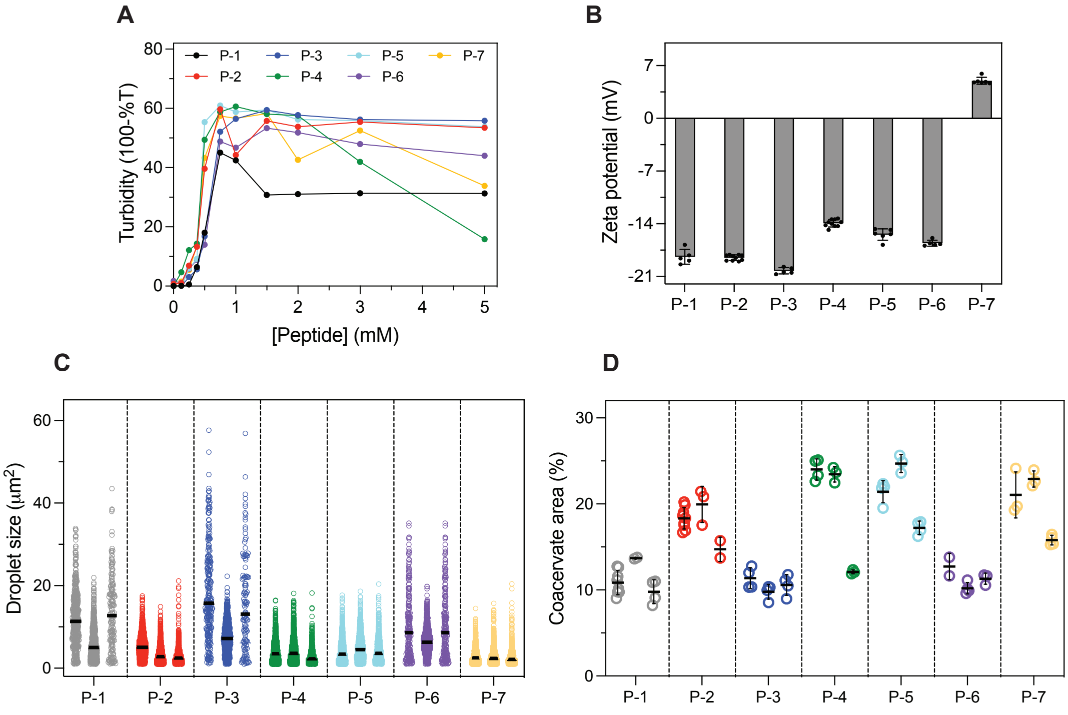

Figure S1: Turbidity, zeta potential and size distribution in coacervate dispersions. A. Turbidity plot measured with fixed HH_{min} concentration (500 μM) and increasing peptide concentration (0 to 5 mM). The absorbance of the solution measured at 500 nm was converted to turbidity and plotted. B. Zeta potential data was obtained for the coacervate microdroplets. Error bar represents the standard deviation from at least three independent measurements (shown). Bar charts are the average of the measurements C. HH_{min}/peptide coacervate droplet size distribution (area) obtained from microscopy images. Violin plots represent the distribution of individual droplet size from three images per experiment from three experimental repeats. Black horizontal lines denote medians of the distribution. D. Total coacervate droplet area (%) in each microscope image frame of respective HH_{min}/peptide coacervate systems obtained from three experimental sets. Each set is an average of multiple image frames from individual experimental repeats. Black horizontal line represents mean values. Error bars represent the standard deviation. Data shows the reproducibility of the experiment. Source data are provided in the repository.

The zeta potential of the droplets (HH_{min}: Peptide; 250 μM:500 μM) was measured with a Zetasizer-nano ZSP (Malvern Instruments Ltd, UK). The zeta potential was determined according to

the manufacturer's protocol using 80 μL dispersions loaded into the disposable folded capillary cell (Malvern, DTS1070). Each sample was measured three times (each measurement is the average of 40 runs) and the mean values and standard deviations were calculated (Figure S1B).

3.2. Supplementary Information Note 1: Discussion of zeta potential data

We used zeta potential measurements to determine the surface properties of the coacervates (Figure S1B). We found that coacervates formed with P-1 to P-6 all had negative zeta potential values with small variations between them, ranging from -13.9 ± 3.4 mV (P-4) to -20.2 ± 3.4 mV (P-3) whilst coacervates formed with P-7 had a positive zeta potential (+4.98 ± 4.0 mV). This was interesting, considering that all coacervates had a 2-fold excess positive charge in coacervate formation; only the P-7 coacervate system showed a positive surface charge from the zeta potential measurement. The peptide with uniform positive charge (P-7) clearly distinguished itself from the remaining peptide coacervates, which had random charge distributions, regardless of the total charge equivalence in the dispersion.

3.3. Discussion of droplet size analysis

Analysis of confocal fluorescence microscopy images showed that the droplet size varies between coacervate systems prepared from the same ribozyme but different peptide sequences (Figure S1C). However, comparison of the droplet size for a given peptide across replicates showed a variability in droplet size distribution. This is most likely due to the fact that droplet size is not a thermodynamic parameter and kinetic effects such as droplet fusion can lead to variation in droplet size depending on the time of imaging. Therefore, we determined the total fraction of area occupied by the coacervates in a single image frame to better compare provide between samples (Figure S1D). We observed limited fluctuations in the total coacervate area fraction among three replicates of each coacervate system and the results showed different peptide sequences lead to different total fraction of area occupied by the coacervates. It is important to note that this confocal microscopy analysis provides a qualitative comparison between the size and area fraction occupied by coacervates.

4.0. Characterisation of RNA and determination of ribozyme kinetics

4.1. Testing ribozyme function in buffer

To test the activity and the quality of the ribozyme in the buffer, all fluorophore-tagged samples (ribozyme and substrate) were prepared in 1X reaction buffer (10 mM Tris + 1 mM $MgCl_2$, pH 8.1) to a final concentration of 0.8 μM in 20 μL of solution for both the ribozyme and substrate. The samples were incubated for 30 min at room temperature and then quenched with 2x loading dye. 4 μL of the sample was loaded into a 20% urea-PAGE gel and ran at 300 V in 1x TBE buffer. The gel was imaged using a Typhoon 9500 Fluo Phospho Imager (GE Healthcare Life Sciences) using either λ_{ex} = 473 nm and λ_{em} = 520 nm or λ_{ex} = 550 nm, λ_{em} = 570 nm (Figure S2).

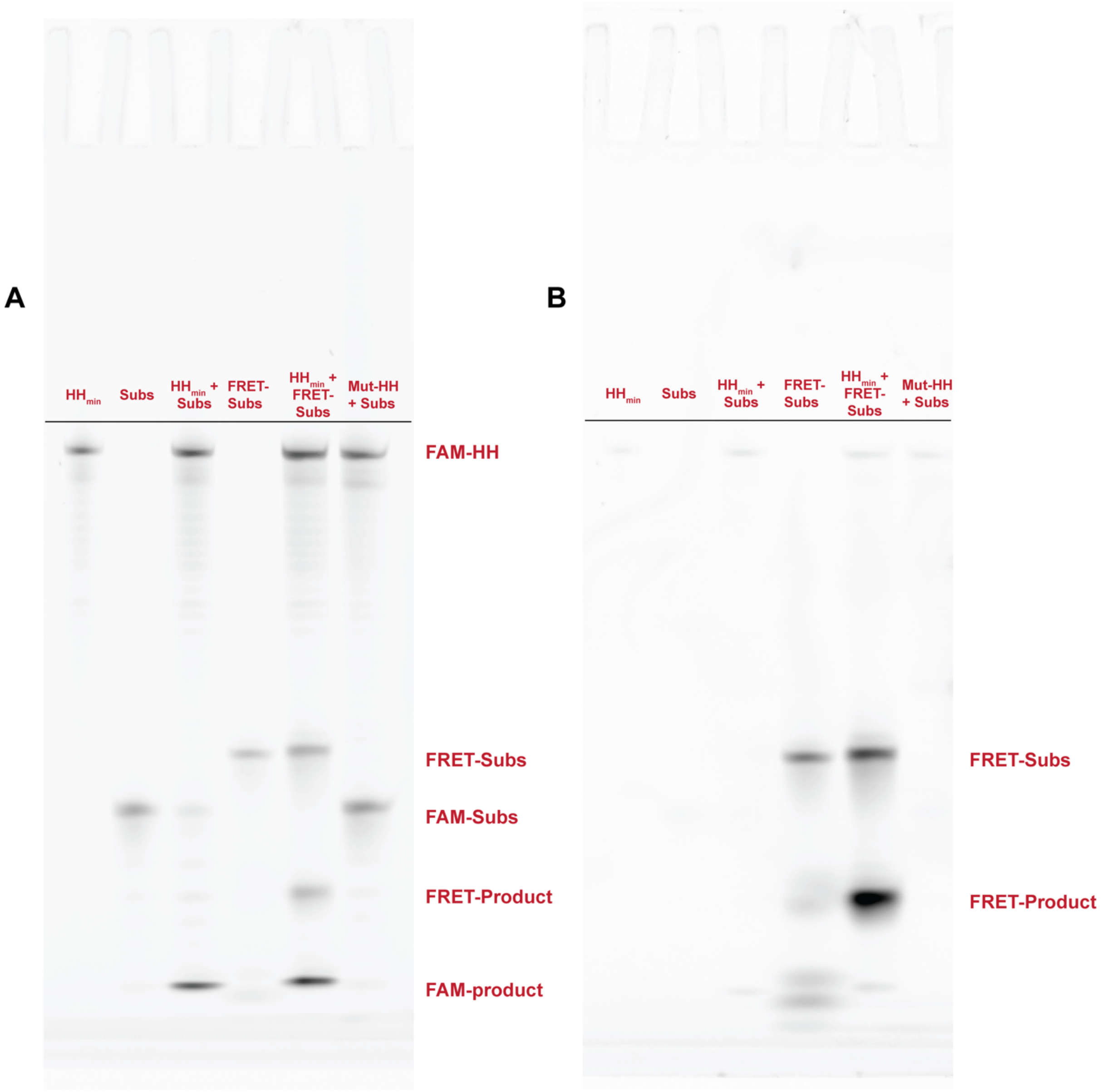

Figure S2: Characterisation of fluorophore-tagged HH_{min} ribozyme (FAM-HH) and substrate (FAM-Subs and FRET-Subs). A. Gel electrophoresis image captured using Cy2 filter. HH_{min} reaction with FRET-subs (with FAM and Alexa-532 dyes) shows two cleaved products, one as an intense FAM-product (with FAM dye) and another with a faint band corresponding to the FRET product (with Alexa 532 dye). Mut-HH does not react with the substrate showing intact FAM-subs. B. The same gel image was imaged using a Cy3 filter to observe Alexa-532 fluorescence intensity. The intense band for FRET-product shows the product tagged with Alexa-532. Other FAM-tagged RNAs were not detectable in this Cy3 filter. Results show that the ribozyme is active and the FRET substrate works as designed.

4.2. Characterisation of ribozyme kinetics after coacervate formation

4.2.1. Characterisation of ribozyme kinetics from the pellet

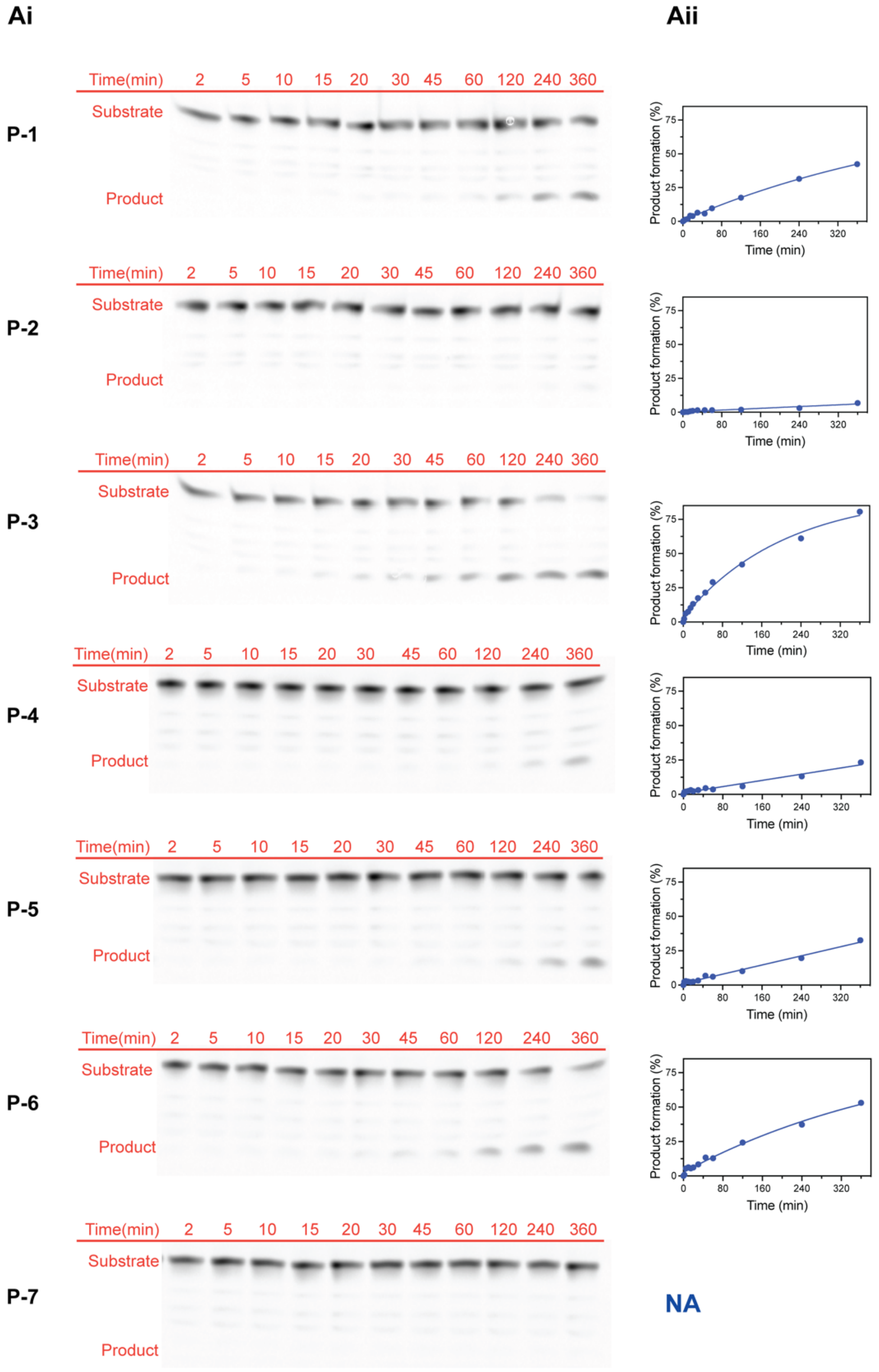

Figure S3: HH_{min} reactivity HH_{min}/peptide from the pellet containing coacervate . Ai. Gel electrophoresis images showing HH_{min} reaction at eleven time points in the presence of the seven different peptides. The fluorescence intensity of the FAM-tagged substrate and the cleaved product was quantified using ImageJ 2.30.051 software. Aii. Plots showing product formation with time, the solid line shows data fitting. Uncropped gels are provided in the data repository and in Supplementary Information section 9.0. Source data are provided in the repository.

4.2.2. Characterisation of ribozyme from the coacervate dispersion

Analysis of ribozyme reactions performed within the coacervate dispersion were treated in the same way as described in the materials and methods only without the centrifugation and supernatant removal steps (Figure S5B).

4.3. Characterisation of ribozyme kinetics in buffer

To obtain the rate constant for HH_{min} in the absence of peptide in buffer solution, HH_{min} (250 µM) and FAM- substrate (50 µM) were mixed in a reaction volume totalling 40 µL in a centrifuge tube. After each time point (2 min, 5 min, 10 min, 15 min, 20 min, 30 min, 45 min and 60 min), 4 µL of the dispersion was withdrawn and quenched with 2x RNA loading dye mixed with 10 mM sodium hexametaphosphate and then run on a 20 % denaturing urea-PAGE gel (Figure S4).

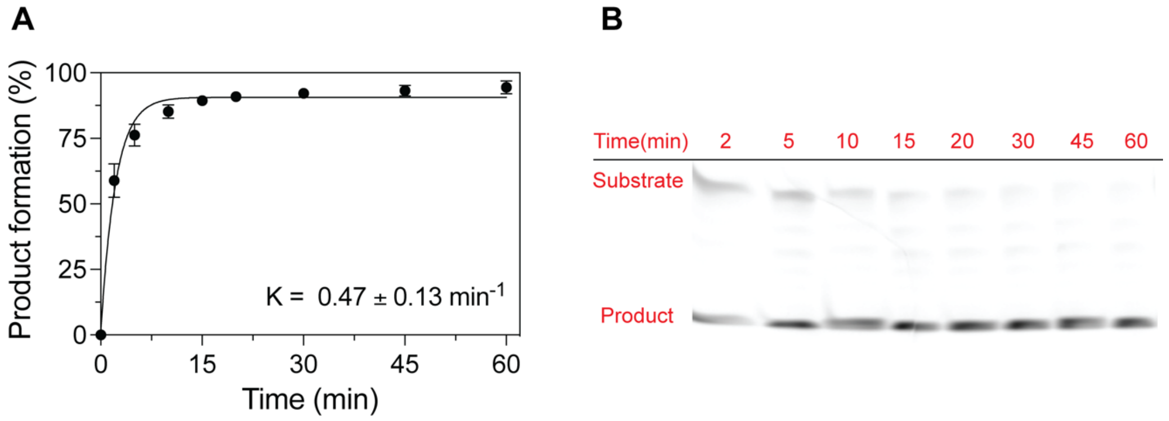

Figure S4: HH_{min} reaction kinetics in the absence of peptides in buffer monitored by gel electrophoresis. A. Product formation (%) plot of 250 µM HH_{min} with 50 µM FAM substrate in buffer. Uncleaved substrate and cleaved product intensity were quantified and product formation (%) was plotted with respect to time. The plot shows an average of three independent reactions and error bars indicate standard deviation. B. Gel image showing substrate and product band fluorescence intensity from the HH_{min} reaction with increasing time. Uncropped gels are provided in the data repository. Source data are provided in the repository.

4.4. Ribozyme reaction in the supernatant

To determine the contribution of ribozyme cleavage from the supernatant to the overall reaction, the reaction mixture was prepared in the following way. Immediately after substrate addition, the mixture was centrifuged and 3 µL supernatant was separated and incubated for 6 hours. This supernatant mixture was quenched as described above and run on a 20 % UREA-PAGE gel along with 3 µL of pellet containing coacervate phase (Figure S5Ai). Both solutions were diluted the same amount. To obtain the ratio of product in the supernatant vs the pellet, the gels were analysed in FIJI to obtain the band intensities of the product in the supernatant and the pellet. The ratio was obtained by dividing the band intensity in the pellet by band intensity of the supernatant (Figure S5Aii).

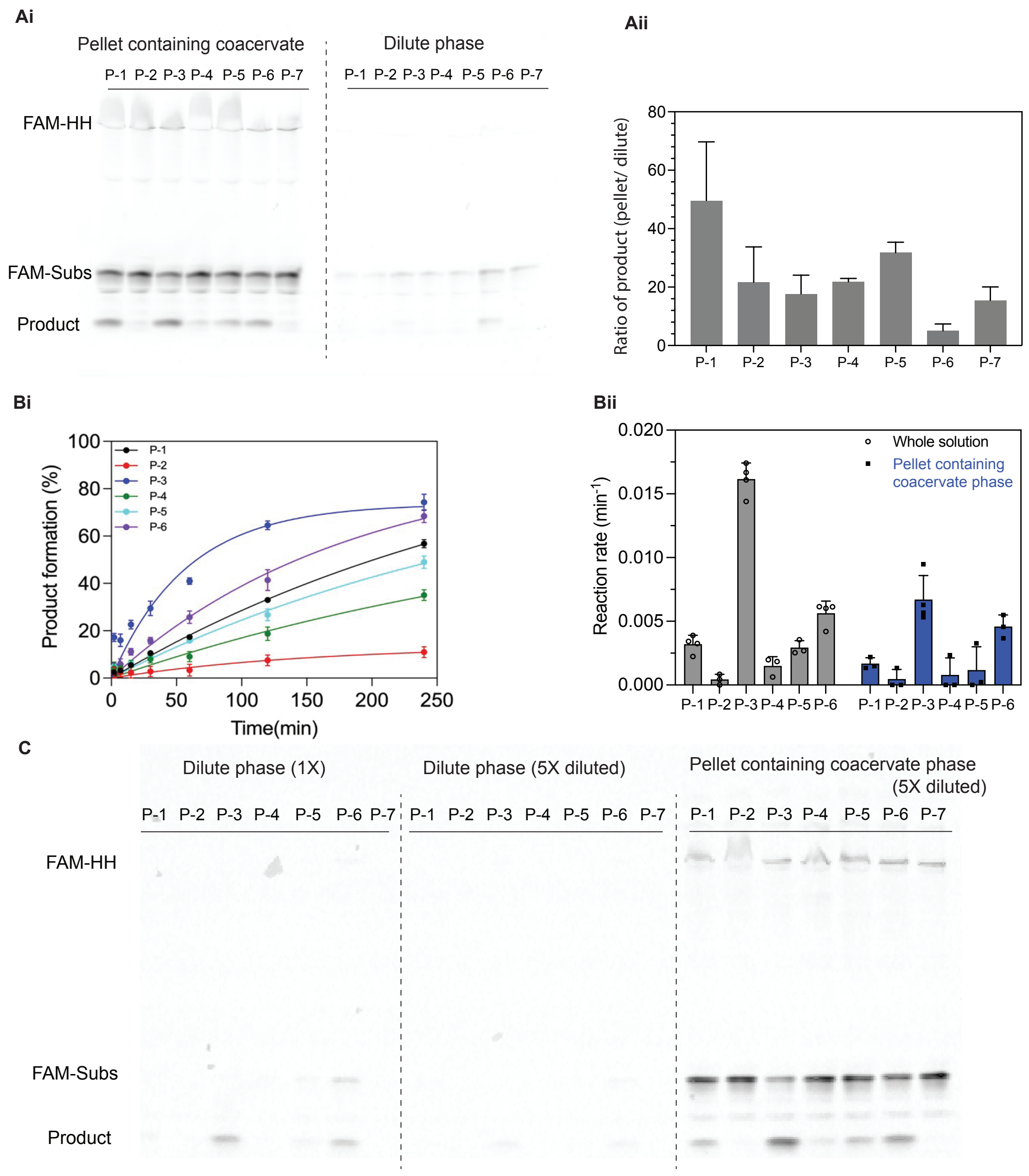

Figure S5: Ribozyme is active within coacervate dispersions. Ai. Gel image showing FAM-labeled contents of the coacervate pellet and supernatant fractions. Here, the supernatant (3 μL) was separated from the coacervate-containing pellet (3 μL) immediately after substrate addition by centrifugation and imaged after 6 hours of incubation Aii. Analysis of the product bands of the pellet containing coacervate (left) and the supernatant (right) using imageJ 2.3.051 provide the ratio of the product (see Supplementary Information Section 4.4). The results show that in all cases there is more product produced within the coacervate-containing pellet compared to the supernatant. Data points are from one sample and error bars are from the analysis. B. (i) Kinetic profiles of product formation in whole dispersions for each of the peptide / RNA systems obtained from gel electrophoresis. (ii) Comparison of rate constants obtained from fits to the dispersion data (i) (grey) with rate constants obtained from 3 μL of pellet containing coacervate (blue) (data from Figure 2). Bar charts are averages, error bars are standard deviation from the individual data points shown (at least 3 in each case). C Gel electrophoresis (images showing HH_{min} reaction from HH_{min}/peptide coacervate systems after 6 hours of incubation. Reaction in the supernatant (left panel) refers to the supernatant separated from the reaction mixture

after centrifugation and mixed with an equal volume of 2x loading dye before running on the gel. The reaction mixture from the dilute phase (5x diluted) (middle panel) and the reaction mixture from the coacervate phase (right panel) were diluted five times before the addition of 2x loading dye and run on the gel. The gel shows negligible concentration of FAM-substrate, product and HH_{min} (with 10% FAM-HH_{min}) in the dilute phase compared to the coacervate phase when diluted the same amount. We observed fluorescence intensity that corresponds to the product in the undiluted supernatant (left panel). Uncropped gels are provided in Supplementary Information Section 9.0. Source data are provided in the repository.

4.5. Determination of RNA fraction in the dilute phase vs the pellet

To measure the ribozyme and substrate concentration inside and outside the coacervate pellet and to compare ribozyme activity in the pellet and supernatant phases, we monitored RNA concentration after 6 hours of reaction using gel electrophoresis. 10% FAM-HH was doped with the HH_{min} (total ribozyme concentration: 250 μM) and mixed with 500 μM of peptides to form droplets. 50 μM FAM-subs was added to start the reaction in a total volume of 10 μL (V^{tot}). After the reaction and quenching with 2X RNA loading dye, the mixture was centrifuged, a 3 μL pellet (V^{pellet}) containing the total coacervate phase and 3 μL of the supernatant were separated and 5-fold diluted with buffer. Another 3 μL of the supernatant (V^{super}) was also collected as undiluted supernatant. All three sets were subjected to gel electrophoresis on 20 % urea-PAGE (Figure S5C).

We are interested in the fraction of product present in the dilute phase given by

$$F_{prod}^{dil} \equiv \frac{N_{prod}^{dil}}{N_{prod}^{tot}} = \frac{N_{prod}^{dil}}{N_{prod}^{cond}+N_{prod}^{dil}} \tag{4.1}$$

The difficulty here is that we can't access N_{prod}^{cond} or N_{prod}^{dil} directly from the gel. We assume that the intensities we measure for the gel bands are proportional to the number of product molecules in the pellet (N_{prod}^{pellet}) and in the 3 μL of the supernatant (N_{prod}^{super}).

Equation 4.1 can be rewritten in terms of these experimentally accessible quantities. Consistent with how the experiment was performed, we recognize that the N_{prod}^{dil} product molecules of the dilute phase are split between three separate fractions of the sample, with $N_{prod}^{dil:A}$ contained in the pellet, $N_{prod}^{dil:B}$ in the 4 μL volume of discarded supernatant, and $N_{prod}^{dil:C} = N_{prod}^{super}$ in the 3 μL volume of retained supernatant that was run on the gel. As a result, we have that

$$N_{prod}^{tot} = N_{prod}^{cond} + N_{prod}^{dil:A} + N_{prod}^{dil:B} + N_{prod}^{dil:C} = N_{prod}^{pellet} + N_{prod}^{dil:B} + N_{prod}^{super}, \tag{4.2}$$

where we used $N_{prod}^{pellet} = N_{prod}^{cond} + N_{prod}^{dil:A}$ to account for the presence of condensed as well as dilute-phase material in the pellet. Recalling that the number of molecules in a volume is related to the average concentration c through $N = cV$ and that the product concentrations in each subset of dilute phase are equal, $c_{prod}^{dil} = c_{prod}^{super}$, we can express $N_{prod}^{dil:B}$ in terms of the measured N_{prod}^{super} as

$$N_{prod}^{dil:B} = c_{prod}^{dil} V^{dil:B} = c_{prod}^{super} V^{dil:B} = N_{prod}^{super} \frac{V^{dil:B}}{V^{super}}. \tag{4.3}$$

With Equations 4.2 and 4.3, the denominator from Equation 4.1 becomes

$$N_{prod}^{tot} = N_{prod}^{pellet} + N_{prod}^{super} \left(\frac{V^{dil:B}}{V^{super}} + 1\right). \tag{4.4}$$

Meanwhile, the numerator from Equation 4.1 can be rewritten as

$$N_{prod}^{dil} = c_{prod}^{dil}V^{dil} = c_{prod}^{super}(V^{tot} - V^{cond}) = N_{prod}^{super}\left(\frac{V^{tot}-V^{cond}}{V^{super}}\right), \tag{4.5}$$

where we used $V^{tot} = V^{cond} + V^{dil}$. Finally, substitution of Equations 4.4 and 4.5 into Equation. 4.1 and reorganizing yields

$$F_{prod}^{dil} = \frac{N_{prod}^{super}(V^{tot}-V^{cond})}{N_{prod}^{pellet}V^{super}+N_{prod}^{super}(V^{dil:B}+V^{super})}. \tag{4.6}$$

Equation 4.6 expresses the fraction of product present in the dilute phase in terms of experimentaly measured parametersl. Since $V^{tot} = 10\ \mu\text{L} \gg V^{cond} \approx 0.001\ \mu\text{L}$ (Table S13), the V^{cond} term can be safely neglected. We note that, although the deviation of k from k_{in} shrinks as F_{prod}^{dil} decreases in some cases, such as the limit described above, this behavior is not retained in some other cases, such as Scenario B. F_{prod}^{dil} provides an estimate of the difference between k and k_{in} and does not provide an absolute value.

4.6. Supplementary Information Note 2: Confirmation of product generation in the dense phase with the Scenario B model

4.6.1. Overview

Due to the intrinsic challenges in measuring the reaction within the coacervate phase alone we opted to use a theoretical approach in combination with our experimental data to indirectly ascertain whether any reaction occurs within the coacervate phase. Here, we mathematically model a hypothetical scenario where the product is exclusively produced in the dilute-phase portion of the pellet fraction of a sample. We refer to this model as "Scenario B" to define its role as an alternative to Scenario A, in which reaction takes place in both phases present in the pellet. We use the Scenario B model primarily to estimate an upper-bound on product generation through reactions restricted to the dilute phase only.

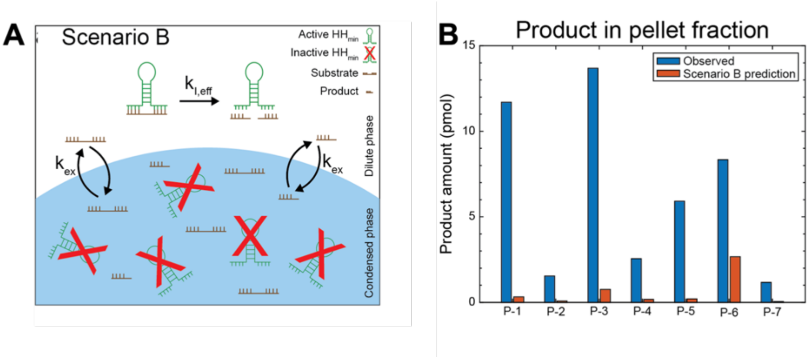

Figure S6: Overview of Scenario B model and key results. A. Schematic illustrating the essential features of the Scenario B model. Condensed-phase HH_{min} is catalytically inactive while HH_{min} in the dilute phase converts substrate to product with rate constant $k_{I,\text{eff}}$. Substrate and product exchange between condensed and dilute phases with rate constant k_{ex}. B. Comparison of product amounts in the pellet observed experimentally (blue, Figure S5Ai) to those predicted by simulation of the Scenario B model after 360 min (orange). In each case, the observed amounts exceed the predictions of Scenario B by several-fold. These data strongly suggest ribozyme is catalytically active in the dense phase.

Within this note, we provide a description of the model and define the key parameters. We then specify how parameter values and initial conditions were determined. We also comment on the rationality of the parameter choices and the approximations made.

In summary, comparing the results from a Scenario B simulation to our experimental results, the amount of product in Scenario B is less in all peptide systems compared to the experimental data (Figure S6). The observed amounts exceed the predictions of Scenario B by several-fold and provide a strong indication that the ribozyme is catalytically active in the coacervate phase.

4.6.2. The Scenario B model

In Scenario B, we model the conversion of substrate into product in the $V_{pellet} = 3$ μL "pellet fraction" obtained following centrifugation of a $V_{tot} = 10$ μL sample immediately upon substrate addition. The pellet fraction is composed of two coexisting phases, the dilute and condensed phases, which we denote by roman numerals I and II, respectively. The volume of the condensed phase is given by

$$V_{II} = \phi_{II} V_{tot}, \tag{4.7}$$

where

$$\phi_{II} = \frac{c^{tot}_{peptide} - c^{I}_{peptide}}{c^{II}_{peptide} - c^{I}_{peptide}} \tag{4.8}$$

is the volume fraction of the condensed phase and is calculated from the concentrations of peptide in each coexisting phase as well as the total peptide concentration averaged over the entire 10 μL sample. The volume of dilute phase in the pellet is

$$V_I^* = V_{pellet} - V_{II}. \tag{4.9}$$

Note that this is distinct from the volume of dilute phase (V_I) in the system as a whole as we consider only 3 μL of the total volume that contains the condensed coacervate phase and dilute phase.

$$V_I = V_{tot} - V_{II}, \tag{4.10}$$

Under Scenario B, the reaction $HH_{min} + Sub \rightarrow HH_{min} + Prod$ is assumed to take place exclusively in the dilute phase. Additionally, substrate and product molecules are assumed to move between phases in order to satisfy the equilibrium partition coefficients for each species i,

$$P_i^{eq} = \frac{c^{II}_{i,eq}}{c^{I}_{i,eq}}. \tag{4.11}$$

Thus, Scenario B models the dynamics of the substrate and product concentrations in each phase (4 concentrations in total) subject to two processes: reaction in the dilute phase and molecular exchange between phases.

Here, we describe these dynamics with a master equation formalism by the following set of 4 ordinary differential equations:

$$\frac{dc^I_{sub}}{dt} = -k_I c^I_{HH_{min}} c^I_{sub} + \frac{\Delta N_{sub}}{V_I^*} k_{ex} \tag{4.12a}$$

$$\frac{dc^{II}_{sub}}{dt} = -\frac{\Delta N_{sub}}{V_{II}} k_{ex} \tag{4.12b}$$

$$\frac{dc^I_{prod}}{dt} = +k_I c^I_{HH_{min}} c^I_{sub} + \frac{\Delta N_{prod}}{V_I^*} k_{ex} \tag{4.12c}$$

$$\frac{dc_{prod}^{II}}{dt} = -\frac{\Delta N_{prod}}{V_{II}} k_{ex}, \qquad (4.12d)$$

where k_I is the reaction rate constant in the dilute phase, $c_{HH_{min}}^{I}$ is the ribozyme concentration in the dilute phase, and k_{ex} characterizes the rate of molecular exchange between phases. k_I, $c_{HH_{min}}^{I}$, and k_{ex} are assumed here to be constant in time. ΔN_{sub} and ΔN_{prod} represent the number of molecules needed to move from the condensed phase to the dilute phase in order to satisfy the equilibrium partition coefficients of each species, and these numbers change with time as the reaction proceeds. At each timepoint, ΔN_{sub} and ΔN_{prod} satisfy the relation

$$\frac{c_i^{II}(t) - \frac{\Delta N_i}{V_{II}}}{c_i^{I}(t) + \frac{\Delta N_i}{V_I^*}} = P_i^{eq}, \qquad (4.13)$$

where i indexes the substrate and product species. Rearranging to solve for ΔN_i gives

$$\Delta N_i = \frac{c_i^{II}(t) - c_i^{I}(t) P_i^{eq}}{\frac{1}{V_{II}} + \frac{P_i^{eq}}{V_I^*}}. \qquad (4.14)$$

Finally, the Scenario B expectation for the number of product molecules in the pellet fraction at time *t* is calculated as $N_{prod}^{Scen.B}(t) = V_I^* c_{prod}^{I}(t) + V_{II} c_{prod}^{II}(t)$.

The dynamics of Equations 4.12a-4.12d were solved numerically via forward integration in MATLAB 2021b using ode45.m given suitable parameter values and initial conditions (Figure S7). Below, we describe our process for selecting parameter values and initial conditions. In most cases, these values reflect constraints obtained directly from experimental design or data.

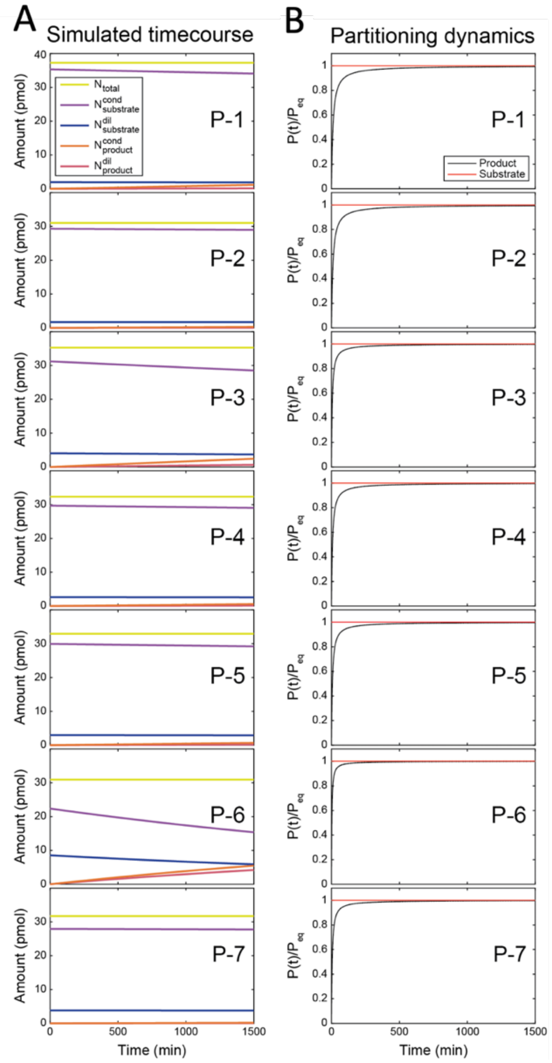

Figure S7: Scenario B model of the kinetics in the pellet fraction. A Simulated timecourses of reaction and partitioning for Scenario B model ($k_{ex} = 1$, other parameters as in Tables S4, S6, and S7) for each peptide/HHmin system. Yellow curve is the sum of the other four. B Partitioning dynamics from the simulations in A. While substrate partitions at equilibrium levels throughout, equilibration of product partitioning proceeds with a delay owing to its initial absence from the system. Source data are provided in the repository.

4.6.3. Determination of parameter values for the Scenario B model

This model for Scenario B includes 7 explicit parameters: k_{ex}, k_I, $c^I_{HH_{min}}$, P^{eq}_{sub}, P^{eq}_{prod}, V^*_I, and V_{II}. We determined numerical values for these parameters as follows, using experimental data when possible.

4.6.3.1. Volumes V^*_I and V_{II}

V_{II} is given by Equations 4.7-4.8 using $V_{tot} = 10$ µL, $c^{tot}_{peptide} = 500$ µM, and $c^I_{peptide}$ and $c^{II}_{peptide}$ as determined from dilute-phase and QPI analysis (as described below), respectively. With V_{II} in hand, V^*_I is given by Equation 4.9 where $V_{pel} = 3$ µL. The volumes V^*_I and V_{II} are reported for each system in Table S4.

Table S4: Volume parameters in Scenario B model

System	V_{II} (μL)	V_I^* (μL)
P-1/HH_{min}	0.00184	2.99816
P-2/HH_{min}	0.00186	2.99814
P-3/HH_{min}	0.00221	2.99779
P-4/HH_{min}	0.00179	2.99821
P-5/HH_{min}	0.00219	2.99781
P-6/HH_{min}	0.00188	2.99812
P-7/HH_{min}	0.00149	2.99851

4.6.3.2. Equilibrium partition coefficient P_{sub}^{eq}

To estimate the equilibrium partition coefficient of the substrate between the dense and dilute phases (Equation 4.11), we analysed the supernatant and pellet gel data from Figure S5A. The basic idea is to estimate the substrate concentration in the condensed phase at early times from the pellet gel using mass conservation, knowledge of phase volumes, and an estimate of the substrate concentration in the dilute phase from gel electrophoresis of the supernatant phase. Given that the pellet contains both condensed phase and dilute phase, it would be unreasonable to estimate P_{sub}^{eq} from the ratio of product in the pellet to product in the supernatant.

First, we measure the integrated intensities (after background subtraction) of the substrate and product bands in each lane of each gel. These values are proportional to the number of molecules of each species present at *t* = 360 min:

$$intI_i^{\alpha} = \theta N_i^{\alpha}, \tag{4.15}$$

where α indexes the pellet and supernatant fractions, i indexes the substrate and product species, $intI$ is the integrated intensity, N is the number of molecules, and θ is the proportionality constant that depends on the fluorophore and imaging conditions. Since both the substrate and product carry the same FAM fluorophore, and since both the pellet and supernatant fractions were imaged on the same gel, θ is the same for all 4 variants of Equation 4.15. As the reaction converts substrate into product, the number of substrate molecules observed at *t* = 360 min is lower than the amount present when the supernatant and pellet fractions were separated at the beginning of the reaction.

We note that, in each case, the number of ribozyme molecules in the supernatant fraction is significantly larger than the number of product molecules observed after 6 h (Table S5). This suggests that product inhibition is unlikely to contribute significantly to the kinetics over this timescale and validates our choice to neglect product inhibition in the model (Equations 4.28a-4.28b) to describe kinetics in the supernatant.

Table S5: Amounts in gel from Fig. S5A[a]

	Pellet (pmol)				Supernatant (pmol)			
System	$N_{HH_{min}}^{pel}$	$N_{sub}^{pel}(360)$	$N_{prod}^{pel}(360)$	$N_{sub}^{pel}(0)$	$N_{HH_{min}}^{super}$	$N_{sub}^{super}(360)$	$N_{prod}^{super}(360)$	$N_{sub}^{super}(0)$
P-1/HH$_{min}$	61.28	25.57	11.70	37.27	0.726	1.661	0.296	1.957
P-2/HH$_{min}$	82.87	29.49	1.54	31.03	0.417	1.648	0.081	1.728
P-3/HH$_{min}$	57.39	21.61	13.69	35.29	1.163	3.353	0.702	4.055
P-4/HH$_{min}$	59.85	29.80	2.55	32.35	0.701	2.485	0.166	2.651
P-5/HH$_{min}$	81.92	27.02	5.91	32.94	1.193	2.801	0.190	2.991
P-6/HH$_{min}$	33.60	22.61	8.33	30.95	3.972	6.187	2.386	8.573
P-7/HH$_{min}$	50.10	30.57	1.16	31.73	1.136	3.758	0.048	3.806

[a] All quantities are listed in units of pmol polymer. Numbers in parenthesis indicate the timepoint (in minutes) to which the value applies. Deviations of the sum $N_{sub}^{pel}(360) + N_{prod}^{pel}(360)$ from $N_{sub}^{pel}(0)$ within a row represent rounding error.

To estimate the number of substrate molecules present at the time of separation, we approximate the number of product molecules at time zero as

$$N_{sub}^{\alpha}(0) \approx N_{sub}^{\alpha}(360) + N_{prod}^{\alpha}(360). \tag{4.16}$$

Given that the reaction proceeds on the hours-timescale and the separation via centrifugation requires only a few minutes, this approximation appears reasonable. Values of $N_{sub}^{\alpha}(0)$ are reported for each system in Table S5.

Next, we estimate the number of substrate molecules present in the condensed phase at early times, $N_{sub}^{II}(0)$. From mass conservation,

$$N_{sub}^{pellet}(0) = N_{sub}^{I^*}(0) + N_{sub}^{II}(0), \tag{4.17}$$

where I^* and II refer to the dilute- and condensed-phase portions of the pellet fraction, respectively. At the time of separation, we assume that the concentration of substrate in the dilute-phase portion of the pellet fraction is equal to the concentration of substrate in the supernatant fraction because both consist of pure dilute phase:

$$\frac{N_{sub}^{I^*}(0)}{V_I^*} \equiv c_{sub}^{I^*}(0) = c_{sub}^{I}(0) = c_{sub}^{super}(0) \equiv \frac{N_{sub}^{super}(0)}{V_{super}}, \tag{4.18}$$

where $V_{super} = 3$ μL is the volume of the supernatant fraction loaded onto the gel. Consequently, the number of molecules in the dilute-phase portion of the pellet fraction is

$$N_{sub}^{I^*}(0) = \frac{V_I^*}{V_{super}} N_{sub}^{super}(0) = \left[1 - \phi_{II}\left(\frac{V_{tot}}{V_{pellet}}\right)\right] N_{sub}^{super}(0). \tag{4.19}$$

Combining Equations 4.17 and 4.19, the number of substrate molecules in the condensed phase can be expressed in terms of the substrate amounts in the pellet and supernatant fractions at early times as

$$N_{sub}^{II}(0) = N_{sub}^{pellet}(0) - \left[1 - \phi_{II}\left(\frac{V_{tot}}{V_{pellet}}\right)\right] N_{sub}^{super}(0). \tag{4.20}$$

Next, we calculate the ratio of substrate concentrations in the condensed and dilute phases at early times. To obtain concentrations, we divide the number of substrate molecules in each phase in

the pellet fraction by the respective volumes of those phases in the pellet fraction. For the condensed phase, this yields

$$c_{sub}^{II}(0) = \frac{N_{sub}^{II}(0)}{V_{II}} = \frac{N_{sub}^{pellet}(0) - \left[1 - \phi_{II}\left(\frac{V_{tot}}{V_{pellet}}\right)\right] N_{sub}^{super}(0)}{\phi_{II} V_{tot}}. \quad (4.21)$$

For the dilute phase, Equation 4.18 gives

$$c_{sub}^{I}(0) = c_{sub}^{super}(0) \equiv \frac{N_{sub}^{super}(0)}{V_{super}}. \quad (4.22)$$

The ratio of substrate concentrations in the two phases at early times is then given by

$$\Omega_{sub} \equiv \frac{c_{sub}^{II}(0)}{c_{sub}^{I}(0)} = \left[\frac{N_{sub}^{pellet}(0)}{N_{sub}^{super}(0)} - 1 + \frac{\phi_{II} V_{tot}}{V_{pellet}}\right]\left(\frac{V_{super}}{\phi_{II} V_{tot}}\right). \quad (4.23)$$

Recalling Equation 4.15, this can also be written explicitly in terms of the integrated intensities measured from the gels as

$$\Omega_{sub} = \left[\frac{intI_{sub}^{pellet}}{intI_{sub}^{super}} - 1 + \frac{\phi_{II} V_{tot}}{V_{pellet}}\right]\left(\frac{V_{super}}{\phi_{II} V_{tot}}\right). \quad (4.24)$$

Values of Ω_{sub} are reported for each system in Table S6.

Table S6: Partition coefficient estimates for substrate and product in Scenario B model[a]

System	Ω_{sub}	Ω_{prod}
P-1/HH_{min}	29396	14698
P-2/HH_{min}	27372	13686
P-3/HH_{min}	10462	5231
P-4/HH_{min}	18788	9394
P-5/HH_{min}	13685	6843
P-6/HH_{min}	4158	2079
P-7/HH_{min}	14749	7375

[a] All values are dimensionless. Partition coefficients for substrate are estimated as equal to the concentration ratios calculated from Equation 4.23. Partition coefficients for product are estimated as ½ the value for substrate in the same system. Deviations of Ω_{prod} from $\Omega_{sub}/2$ represent rounding error.

Finally, we use this concentration ratio as an estimate for the equilibrium partition coefficient of the substrate between the two phases:

$$P_{sub}^{eq} \cong \Omega_{sub}. \quad (4.25)$$

This approximation is best in the limit where the timescale for substrate partitioning to equilibrate is shorter than the timescale for the separation of the pellet and supernatant fractions, and both are much shorter than the characteristic timescale of the reaction, i.e.

$$\tau_{\text{partitioning}} < \tau_{\text{centrifugation}} \ll \tau_{\text{reaction}}. \quad (4.26)$$

In our systems, we anticipate that $\tau_{\text{partitioning}}$ is limited primarily by the rate of diffusion in the condensed phase. Our FRET experiments (Figure 3) demonstrate that this timescale varies between different peptide/HH_{min} systems and can be on the order of 10s of minutes. This indicates that substrate partitioning may not yet have equilibrated by the time the supernatant and pellet fractions are separated. Since the substrate is predominantly in the dilute phase upon addition to the sample, Ω_{sub} will lie below the true value of P_{sub}^{eq} in the slow-partitioning limit. Intuitively, this is because the concentration of

substrate in the condensed phase begins near zero and not enough time has elapsed for it to rise to the equilibrium level throughout the entire condensed phase.

To assess the impact of deviations of the partition coefficients on our conclusions, we ran simulations with 10-fold higher partitioning (Figure S8). In each case, we found that increasing the partition coefficient reduces the amount of product generated through Scenario B. So, while our estimate of P may lie below the true equilibrium partition coefficient, using a more accurate (i.e. larger) value further reduces product formation relative to our prediction. In this sense, our prediction is an upper-bound on the contribution of Scenario B to overall production.

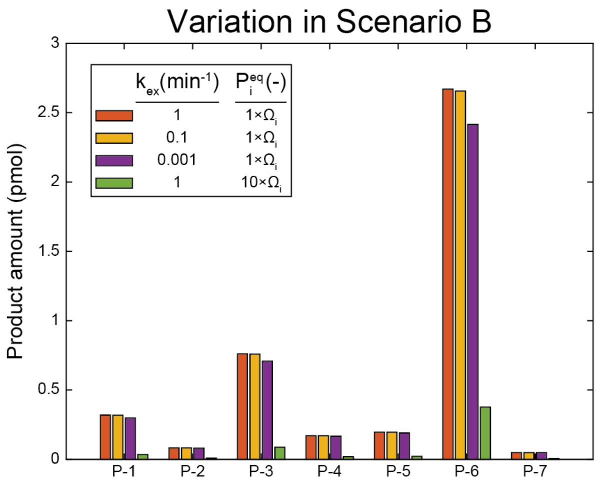

Figure S8: Sensitivity of Scenario B model output to parameters. Comparison of product predictions after 360 min from Scenario B models upon variation of the least-certain model parameters: the exchange rate (k_{ex}) and equilibrium partition coefficients (P_i^{eq}) for the substrate and product. Partition coefficients are quoted as multiples of the concentration ratios between pellet and supernatant fractions, Ω, values of which are listed in Table S6. The model shown previously in Figure S6B with $k_{ex} = 1$ and $P_i^{eq} = \Omega_i$ (red-orange) is reproduced here for comparison. For fixed partition coefficient, product amounts decrease slightly as the exchange rate is reduced 1000-fold. For fixed exchange rate, product amounts decrease ~8-fold upon 10-fold increase in equilibrium partition coefficient. These results suggest that revising parameter values to more accurately reflect the timescale of partitioning will tend to reduce the generation of product under Scenario B. In this sense, the parameters used in Figure S7 serve as an upper-bound on product generation under Scenario B. Source data are provided in the repository.

4.6.3.3. Equilibrium partition coefficient P_{prod}^{eq}

Estimates of the equilibrium partition coefficient for the product are given by

$$P_{prod}^{eq} \cong \frac{\Omega_{sub}}{2}, \qquad (4.27)$$

and are reported for each peptide/HH$_{min}$ system in Table S6. This choice (Equation 4.27) is motivated by the observations that 1) the partition coefficient generally increases with chain length for strong polyelectrolytes in coacervate-forming systems[4,5], and 2) the product is about half the size of the substrate. We emphasise that we use Equation 4.27 to provide a qualitative estimate of the relationship between the partitioning of the substrate and product. While Equation 4.27 may not be accurate quantitatively, we expect that the qualitative relationship is correct. We note that the particular choice

of P_{prod}^{eq} does not impact the conclusions we draw from the Scenario B model. This is because the reaction rate is assumed to be independent of the local product concentration, (i.e. no product inhibition). As a result, the reaction terms in Equations (4.12a-4.12d) are independent of local product concentrations and therefore independent of P_{prod}^{eq}. Since the number of ribozyme molecules in the dilute-phase portion of the pellet is, in most cases, less than the total amount of product observed within the pellet fraction (Table S5), product inhibition could influence the rate of product formation in the dilute phase. However, we note that incorporating product inhibition into the model would further reduce the product generated by Scenario B and would not increase it. Neglecting product inhibition is thus consistent with our use of the Scenario B model to place an upper-bound on product formation in the dilute-phase portion of the pellet fraction.

4.6.3.4. Effective rate constant $k_{I,\text{eff}} = k_I \times c_{HH_{min}}^I$

Note that the parameters k_I and $c_{HH_{min}}^I$ appear in the model only as the product $k_I c_{HH_{min}}^I = k_{I,\text{eff}}$. It is therefore sufficient to specify this product alone, from which the rate constant k_I can be determined using measured values of $c_{HH_{min}}^I$ (Figure 4B) if desired.

For each peptide/HH_{min} system, we estimate the effective dilute-phase rate constant $k_{I,\text{eff}}$ by fitting a minimal kinetic timecourse of product formation in the supernatant fraction. We assume that the supernatant fraction is a homogeneous solution without any dense phase. We model the HH_{min}-catalyzed conversion of substrate to product in the homogeneous supernatant fraction as a single-step reaction governed by the master equations

$$\frac{dc_{sub}^{super}}{dt} = -k_I c_{HH_{min}}^I c_{sub}^{super} = -k_{I,\text{eff}} c_{sub}^{super} \tag{4.28a}$$

$$\frac{dc_{prod}^{super}}{dt} = +k_I c_{HH_{min}}^I c_{sub}^{super} = +k_{I,\text{eff}} c_{sub}^{super}. \tag{4.28b}$$

Following integration, this pair of equations has the simple set of solutions

$$c_{sub}^{super}(t) = c_{sub}^{super}(0) e^{-k_{I,\text{eff}} t} \tag{4.29a}$$

$$c_{prod}^{super}(t) = c_{prod}^{super}(0) + c_{sub}^{super}(0)[1 - e^{-k_{I,\text{eff}} t}]. \tag{4.29b}$$

We specify the initial conditions by approximating the product concentration at the time the supernatant fraction is separated from the pellet fraction to be zero,

$$c_{prod}^{super}(0) = \frac{N_{prod}^{super}(0)}{V_{super}} \approx 0, \tag{4.30}$$

implying via Equation 4.16 that

$$c_{sub}^{super}(0) = \frac{N_{sub}^{super}(0)}{V_{super}} \approx \frac{N_{sub}^{super}(360) + N_{prod}^{super}(360)}{V_{super}}. \tag{4.31}$$

Finally, we determine $k_{I,\text{eff}}$ by fitting the normalized product concentration

$$y(t) = c_{prod}^{super}(t) / c_{sub}^{super}(0) \tag{4.32}$$

at two timepoints, $t = 0$ (assumed zero) and 360 min (determined from the gel), to the normalized version of Equation 4.29b with the initial conditions (Equations 4.30-4.31)

$$y(t) = [1 - e^{-k_{I,\text{eff}} t}]. \tag{4.33}$$

The fits are shown in Figure S9 and the values of $k_{I,\text{eff}}$ obtained through this fitting procedure are reported in Table S7.

We note that, in each case, the number of ribozyme molecules in the supernatant fraction is significantly larger than the number of product molecules observed after 6 h (Table S5). This suggests that product inhibition is unlikely to contribute significantly to the kinetics over this timescale, and validates our choice to neglect product inhibition in the model used to describe kinetics in the supernatant (Equations 4.28a-4.28b).

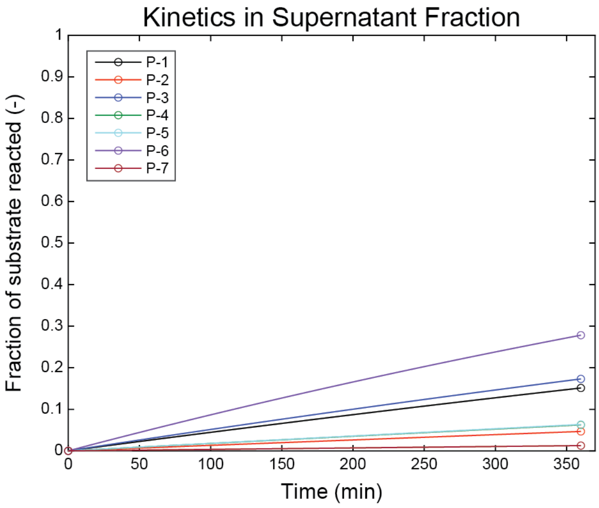

Figure S9: Fit to kinetics in supernatant fraction for Scenario B model. Minimal time-courses (circles) constructed from electrophoresis measurements at t = 360 min (Figure S5A, Table S5) and the assumption of zero product at t = 0. Solid curves represent fits to Equation 4.33. The only free parameter is the effective reaction rate constant, whose values are reported in Table S7.

Table S7: Summary of fits to reaction kinetics in supernatant used in Scenario B model[a]

System	$k_{I,\mathrm{eff}}$ (min^{-1})	$\delta k_{I,\mathrm{eff}}$ (min^{-1})
P-1/HH_{min}	4.555E-04	1.140E-08
P-2/HH_{min}	1.328E-04	1.275E-08
P-3/HH_{min}	5.280E-04	2.700E-10
P-4/HH_{min}	1.796E-04	1.840E-07
P-5/HH_{min}	1.822E-04	4.552E-09
P-6/HH_{min}	9.061E-04	3.975E-09
P-7/HH_{min}	3.525E-05	3.911E-07

[a] As each fit is to a two-point time-course, the fit Adj. R^2 = 1 in each case. Correspondingly, the quoted uncertainty in effective dilute-phase rate constant, $\delta k_{I,\mathrm{eff}}$, reflects numerical precision of the fitting rather than statistical uncertainty.

4.6.3.5. Constant characterising rate of molecule exchange between phases, k_{ex}

This is a phenomenological parameter used to characterize the partitioning process in a simple way. In general, we expect that the timescale for a molecular species to equilibrate concentrations between two phases depends on the size distribution of coacervate droplets present in the sample as well as the diffusion coefficient of the species in the coacervate phase. For simplicity, we do not attempt to model these dependencies explicitly or to account for system-specific differences in the exchange kinetics beyond the level of differences in the total volume of condensed phase. Rather, we set a

relatively fast value of $k_{ex} = 1/\text{min}$ as a default value for all peptide/HHmin systems. Depending on the system, this value is ~1000-fold to 30,000-fold larger than $k_{I,\text{eff}}$, corresponding to a limit in which $\tau_{\text{partitioning}} \ll \tau_{\text{reaction}}$. As mentioned above, we observe the partitioning timescale can be on the order of 10s of minutes (Figure 3), suggesting that a rate closer to that of $k_{I,\text{eff}}$ may be more accurate.

To assess the impact of the fast exchange assumption on our conclusions, we also ran simulations with 10-fold and 1000-fold lower values of k_{ex}. In each case, we found that decreasing k_{ex} reduces the amount of product generated through Scenario B (Figure S8). We note that the dependence on k_{ex} of the product amount generated at *t* = 6 h is very weak. Over the range of parameters examined here, this indicates that the amount of product generated through Scenario B is much less sensitive to the choice of k_{ex} than to the choice of P_{sub}^{eq} (Figure S8). Taken together, these observations indicate that our choice of $k_{ex} = 1/\text{min}$ is again consistent with our use of the Scenario B model to place an upper-bound on product generation in the dilute-phase portion of the pellet fraction.

4.6.4. Determination of initial conditions for the Scenario B model

To be fully-specified, the Scenario B model given in Equations 4.12a-4.12d requires initial conditions for each of the four dynamical variables c_{sub}^{I}, c_{sub}^{II}, c_{prod}^{I}, and c_{prod}^{II}. Consistent with the approximations used to determine parameter values and discussed above, we approximate the number of product molecules in the pellet at time *t* = 0 to be zero, such that Equation 4.16 holds. $c_{sub}^{II}(0)$ and $c_{sub}^{I}(0)$ are thus given by Equations 4.21 and 4.22, respectively, while $c_{prod}^{I}(0) = c_{prod}^{II}(0) = 0$.

4.6.5. Conversion between integrated intensity on the gel and number of molecules, θ

Aside from assessing the potential impact of product inhibition, none of the analysis described above nor any conclusions drawn from it require precise knowledge of θ, defined in Equation 4.15. In particular, the Scenario B master equations are readily re-written in terms of dynamical integrated intensities instead of concentrations and lead to equivalent conclusions.

That said, we chose to report amounts in units of picomole rather than integrated intensity owing to the former's clear physical meaning and potential transferability beyond the current work. We determined θ for both HHmin and substrate as

$$\theta_i = \frac{\langle intI_i^{tot} \rangle_n}{c_i^{tot} V_{tot} \left(q_i^{tot}\right)^{-1}} \tag{4.34}$$

where c_i^{tot} is the total concentration of species i present in the system (in units of charge equivalents; 50 μM for substrate, 250 μM for HHmin), q_i^{tot} is the formal charge of species i, $intI_i^{tot}$ is the total integrated intensity expected for the species in the entire system at t = 0 given as

$$intI_i^{tot} = \begin{cases} intI_{HH_{min}}^{pellet} + \left(\frac{V_{tot}-V_{pellet}}{V_{super}}\right) intI_{HH_{min}}^{super}, \text{for } i = HH_{min} \\ \left(intI_{sub}^{pellet} + intI_{prod}^{pellet}\right) + \left(\frac{V_{tot}-V_{pellet}}{V_{super}}\right)\left(intI_{sub}^{super} + intI_{prod}^{super}\right), \text{for } i = sub \end{cases} \tag{4.35}$$

and $\langle \ldots \rangle_n$ denotes an average over the $n = 7$ peptide/HHmin systems. We found $\theta_{sub} = 39.3 \pm 4.8$ and $\theta_{HH_{min}} = 5.7 \pm 1.4$. The ~10-fold lower value for $\theta_{HH_{min}}$ reflects the different fractions of FAM-labeled molecules in the experiment ($f_{HH_{min}}^{label} = 0.1$, while $f_{sub}^{label} = 1$).

4.7. Detection of substrate and product with the FRET-substrate using confocal microscopy

Droplets were formed with 250 µM HH_{min} and 500 µM peptide in the reaction buffer and loaded into ibidi chambers at 25°C. After droplet settling by gravity, 50 µM of FRET-subs was added to the corner of the well and gently mixed without touching the surface of the slide. Immediately after addition, confocal Z-stack images (0.3-µm step size, 15 slices) were acquired in a time-lapse series with 1 min intervals for 30-60 min with λ_{ex} = 488 nm. To minimise the crosstalk between the two FRET dyes (FAM and Alexa532), the emission spectral range was narrowed down to λ_{em2} = 490-509 nm (Em2) (for FAM) and λ_{em1} = 614-660 nm (Em1) (for Alexa532) (Figure S10). Images were processed using ImageJ 2.3.051 and fluorescence intensities were plotted using GraphPad Prism 9.4 (Figures S11 and S12).

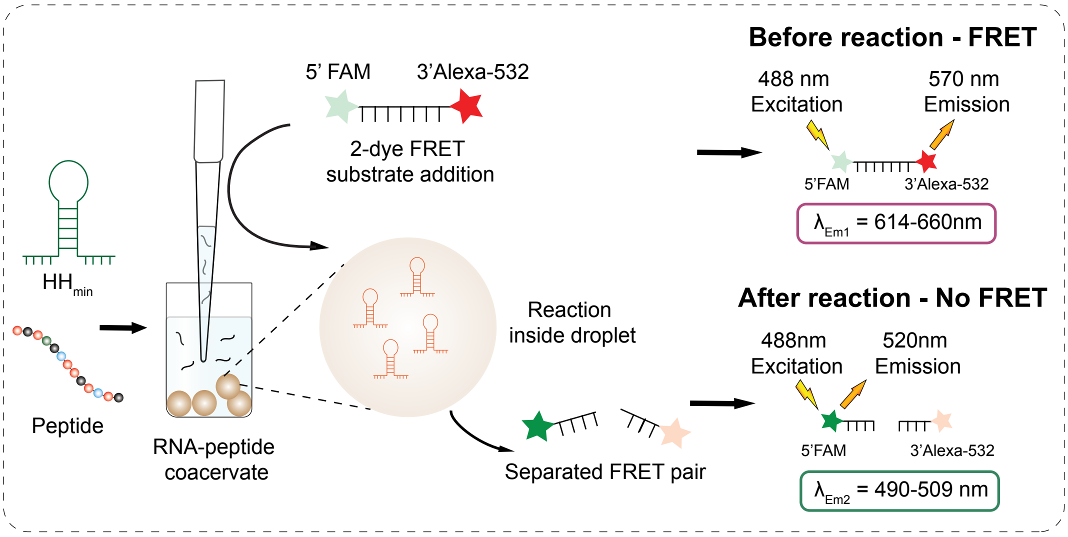

Figure S10: Design of FRET experiment. Schematic representation showing HH_{min}/peptide droplet reaction with FRET substrate measured using microscopy. FRET substrate was added to the dispersion and localisation to the coacervate droplet was observed through fluorescence microscopy. Upon excitation with 488 nm, the substrate was observed via λ_{Em1} (red channel) and the cleaved product was observed through λ_{Em2} (green channel).

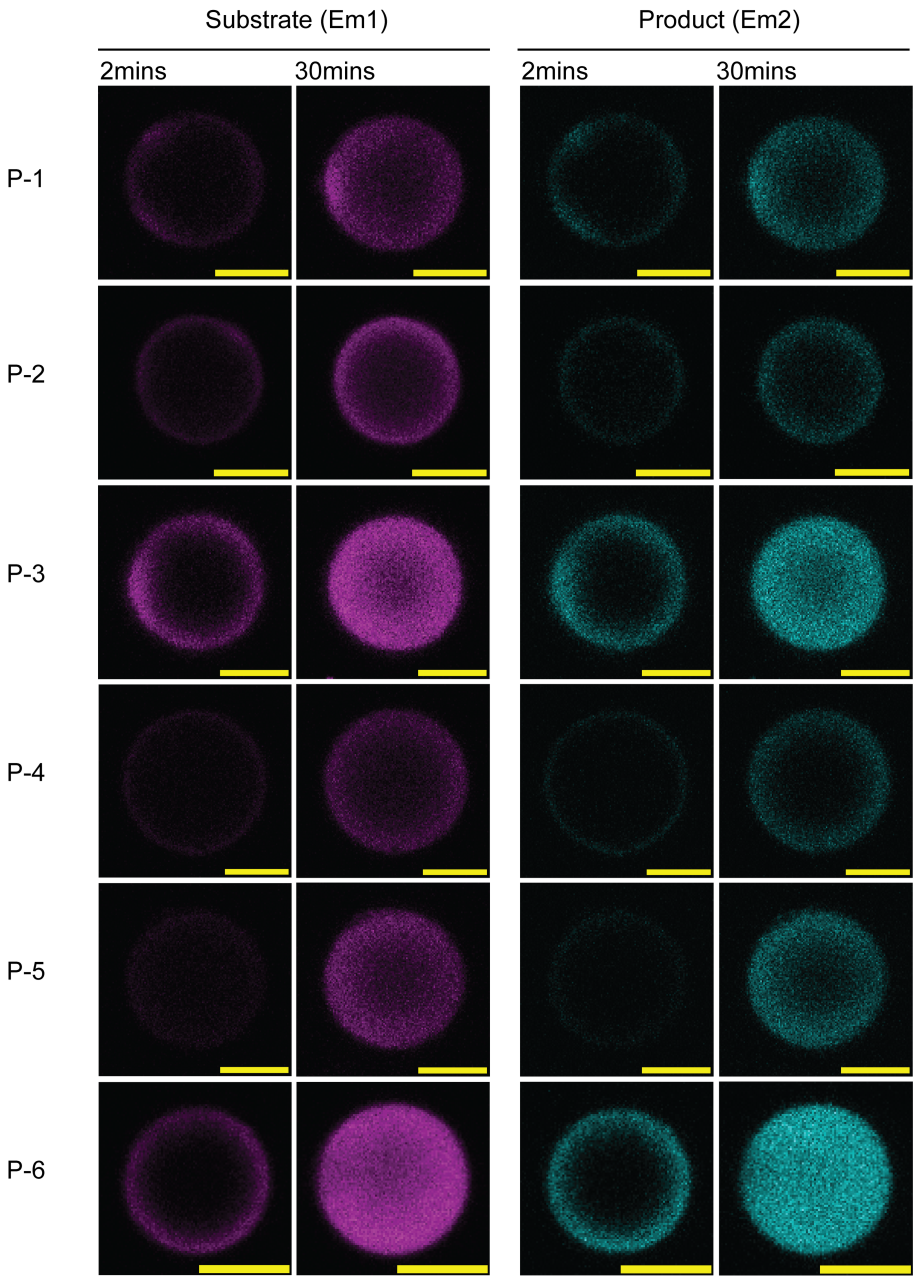

Figure S11: Monitoring FRET-substrate and product in peptide/HH$_{min}$ coacervate droplets. Representative confocal microscopy images from at least three repeats showing the distribution of FRET-substrate and product after 2 and 30 min for each of the coacervate systems prepared with peptides (P-1 to P-6) and HH$_{min}$ ribozyme. Scale bar: 10 μm.

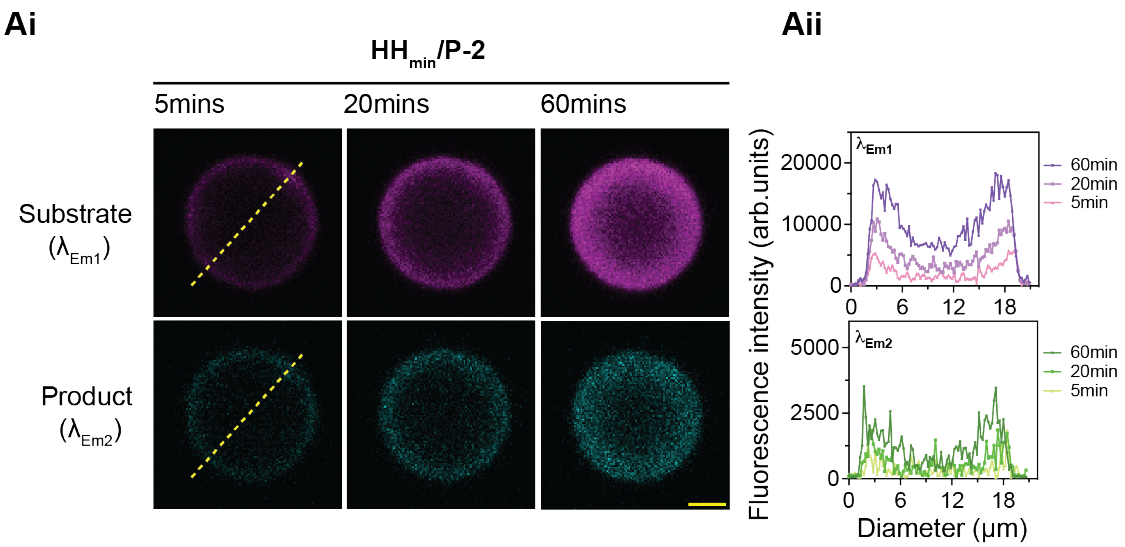

Figure S12: FRET-substrate and product in P-2/HH$_{min}$ coacervate droplet. Ai. Confocal microscopy images show FRET-substrate localisation λ_{Em1} inside HH$_{min}$ / P-2 coacervate droplets and cleaved product formation (λ_{Em2}) after 60 min. Aii. The corresponding fluorescence intensity values (across the yellow dashed line) of the droplets show substrate and product intensity at 5, 20 and 60 min. Scale bar: 5 µm. Source data are provided in the repository.

5.0. Determination of tie-lines and HH$_{min}$ and peptide concentrations at phase coexistence

5.1 Overview

To determine the concentration of HH$_{min}$ and peptide within the coacervate phase, we employed an approach based on thermodynamic and optical considerations[6]. This method uses a tie-line analysis in combination with quantitative phase imaging (QPI) and makes it possible to obtain the concentrations of RNA and peptides within the coacervate phase.

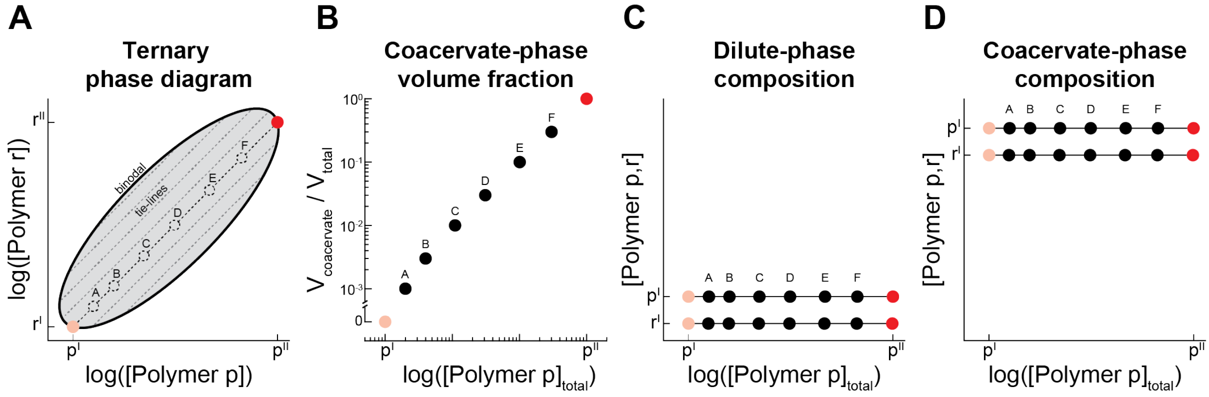

Figure S13: Generic expectations for associative phase separation in a ternary mixture. A. Bold line denotes the binodal, the concentrations at which the system transitions from the one-phase region (homogeneous solution, white) to a two-phase region (phase coexistence, shaded). Within the two-phase region, tie-lines (dotted lines) connect the average system composition (e.g. point A) to the concentrations in the coexisting phases (dilute phase in pink, condensed phase in red). B. As one proceeds along the tie-line from A to F, the condensed phase occupies an increasing fraction of the total system volume. Note that the coacervate-phase volume fraction was calculated from the phase diagram by the lever rule for p^{II}/p^{I} = 1000. C. At each point along the tie-line, the concentrations of p

and r in the dilute phase remain constant. D. At each point along the tie-line, the concentrations of p and r in the condensed phase remain constant.

5.2. Determination of tie-lines

For each peptide/HH_{min} system, we determined the tie-line in the two-phase region of the corresponding phase diagram from two points. The first is represented by the total concentrations of peptide c_p^{tot} and ribozyme c_r^{tot} used for our kinetic experiments (500 μM peptide and 250 μM ribozyme). We denote this point on the phase diagram as (c_p^{tot}, c_r^{tot}) and note that it corresponds to the concentrations of peptide and ribozyme averaged over the entire system. The second point corresponds to the dilute-phase concentrations of peptide c_p^{dil} and ribozyme c_r^{dil} and is denoted on the phase diagram as (c_p^{dil}, c_r^{dil}). We discuss measurement of (c_p^{dil}, c_r^{dil}) by standard calibration methods in Section 5.2.1 and computation of tie-line parameters in Section 5.2.2.

5.2.1. Dilute-phase HH_{min} and peptide concentration measurements

10% FAM-HH (25 μM) was mixed with 90% HH_{min} (225 μM) to form droplets with 500 μM peptides. FAM-HH concentration was limited to 10% to keep the total fluorophore concentration, below 1 μM. In 40 μL of buffer solution, all these three components were mixed and droplets were settled for 30 min in centrifuge tubes. The tubes were then centrifuged for 2 min at high speed (~4700 x g) and 20 μL of solution was withdrawn as supernatant for dilute-phase HH_{min} and peptide concentration measurements.

For each peptide's dilute-phase concentration measurement, individual standard curves were prepared using a BCA kit following the manufacturer's protocol. To get the standard curve, a number of peptide solutions with increasing charge concentration (0 to 375 μM) in 20 μL buffer were prepared and mixed with 150 μL BCA working reagent (mixture of Reagent A and B) and incubated for 1 hour in the dark. Dilute-phase solution (20 μL) was also mixed with BCA working reagent exactly at the same time. From these 170 μL mixtures, 50 μL was transferred to 384 well plates in replicates and absorbance was measured at 562 nm using a Tecan Spark 20M microplate reader. The dilute-phase peptide concentration (Figure 4B) was interpolated from the standard curves.

To generate the HH_{min} standard curve, a number of FAM-HH samples were prepared with increasing concentration (molar FAM concentration) from 0 to 320.5 nM in 20 μL buffer solution. In a 384-well plate, the fluorescence intensity of these solutions was measured (λ_{Ex} = 488 nm / λ_{Em} = 520 nm) using a Tecan Spark 20M microplate reader. Data were plotted using Prism and dilute-phase HH_{min} concentration (Figure 4B) was interpolated from the standard curve.

5.2.2. Determination of tie-line parameters

As mass conservation requires that tie-lines are linear functions of concentration for each species[6], measurement of two points on the tie-line in the ([peptide], [HH_{min}])-plane suffice to specify the tie-line fully (see Figure 4A). For the first point, we used the average concentration of each component in the entire system (averaged across both phases) that had been used for our previous kinetic experiments: (c_p^{tot} = 500 μM, c_r^{tot} = 250 μM). This is the same for each peptide/HH_{min} pair. For

the second point, we used the concentrations of peptide and RNA measured in the dilute phase (described in Section 5.2.1). With these two points in hand, a mathematical constraint equation for the tie-line is obtained by linear interpolation. The tie-line parameters determined for each peptide/HH_{min} system are summarized in Table S8.

Table S8: Tie-line parameters

Peptide	m_{TL} (-)	Error (-)[a]	*Y* intercept, b (μM)	Error (μM)[a]
P-1	0.622	0.102	-61.0	-10.0
P-2	0.847	0.349	-173.3	-71.5
P-3	0.899	0.187	-199.6	-41.6
P-4	0.904	0.341	-202.1	-76.3
P-5	0.888	0.241	-194.1	-52.7
P-6	0.588	0.072	-43.9	-5.4
P-7	0.882	0.476	-191.0	-103.1

[a] Errors represent propagation of uncertainty from the dilute-phase and average concentrations.

5.3. Determination of condensed-phase HH_{min} and peptide concentrations

To determine the concentration of HH_{min} and peptide within the coacervate phase, we employed a novel label-free approach[6]. The method is based on the physical requirement of equilibrated two-phase systems to satisfy specific thermodynamic and optical constraints. The condensed-phase concentrations of peptide (c_p^{cond}) and ribozyme (c_r^{cond}) are given in terms of the readily measurable quantities Δn, $\frac{dn}{dc_p}$, $\frac{dn}{dc_r}$, c_p^{dil}, c_r^{dil}, c_p^{tot}, and c_r^{tot} by

$$\begin{bmatrix} c_p^{cond} \\ c_r^{cond} \end{bmatrix} = \begin{bmatrix} \frac{\Delta n}{-det(M)} + c_p^{dil} \\ \frac{\Delta n m_{TL}}{-det(M)} + c_r^{dil} \end{bmatrix}, \tag{5.1}$$

where

$$det(M) = -\frac{dn}{dc_p} - m_{TL}\frac{dn}{dc_r} \tag{5.2}$$

and

$$m_{TL} = \left(\frac{c_r^{tot} - c_r^{dil}}{c_p^{tot} - c_p^{dil}}\right). \tag{5.3}$$

Δn is the refractive index difference between the dense and dilute phases. $\frac{dn}{dc_p}$ and $\frac{dn}{dc_r}$ are the refractive index increments of peptide and ribozyme. c_p^{dil} and c_r^{dil} are the dilute-phase concentrations of peptide and ribozyme. c_p^{tot} and c_r^{tot} are the total concentrations of peptide and ribozyme averaged over the entire system. Graphically, Equation 5.1 corresponds to the intersection of a tie-line with an isorefractive line, where the tie-line slope is given by Equation 5.3 and the slope of the isorefractive line is given by $m_{IRL} = -\frac{\frac{dn}{dc_p}}{\frac{dn}{dc_r}}$.

5.3.1. Error analysis and propagation

The concentrations in the condensed phase were determined from Equation 5.1, with Equations 5.2 and 5.3. Together, these equations contain the seven known parameters Δn, $\frac{dn}{dc_p}$, $\frac{dn}{dc_r}$, c_p^{dil}, c_r^{dil}, c_p^{tot}, and c_r^{tot}. Associated with each parameter x is an uncertainty σ_x. Therefore, the uncertainty of the condensed-phase concentration was determined as previously described[6] by propagating the uncertainties from each of the variables using Jacobians. For each species, the variance of c_i^{cond} was calculated as

$$\left(\delta c_i^{cond}\right)^2 = J_i \Sigma^x J_i^T, \tag{5.4}$$

where J_i is the Jacobian of the function c_i^{cond}, J_i^T is its transpose, and Σ^x is a square matrix with matrix elements

$$\Sigma_{jk}^x = \rho_{jk} \sigma_j \sigma_k, \tag{5.5}$$

where ρ_{jk} represents the cross-correlation between the uncertainties in the parameters x_j and x_k. As each of the seven parameters were determined independently, all non-diagonal elements of the cross-correlation weight matrix ρ_{jk} were set to zero. The final reported uncertainty on condensed-phase concentrations is the square root of the variance.

5.4. Supplementary Information Note 3: Implications from comparative tie-line analysis

While charge concentration units are commonly used for polyelectrolyte-complex coacervates formed from charged homopolymers, mass or volume concentrations are more commonly used for heteropolymeric systems where interactions between non-charged monomers may contribute. By measuring tie-lines and coacervate composition, we found that the tie-lines for the 7 systems examined here collapse into two clear families when plotted in units of charge concentration (Figure 4), whereas the tie-lines for the different systems appear unrelated in units of mass concentration (Figure S14).

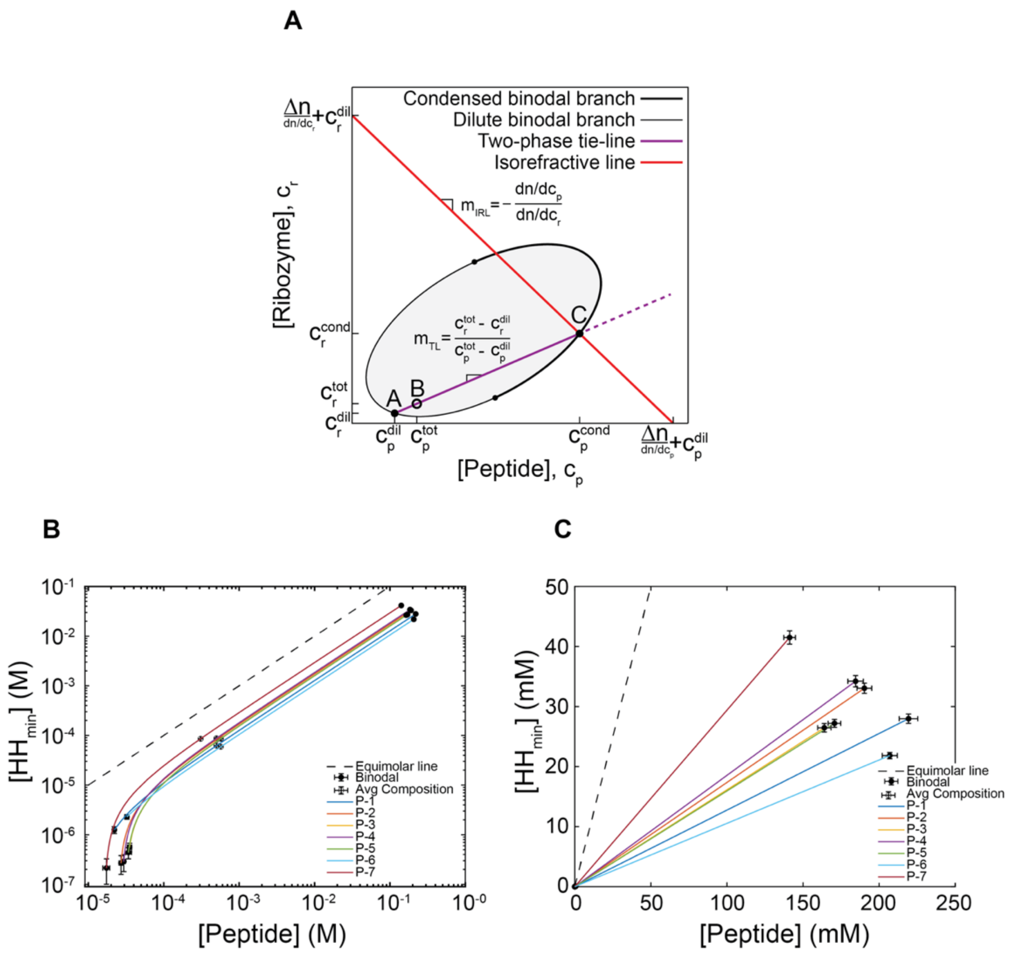

Figure S14: Tie-lines presented in units of polymer concentration. A. Schematic representation (adapted from [6]) showing the geometry of a ternary phase diagram for determination of polymer concentrations in the coacervate. The method obtains the concentrations (c_p^{cond}, c_r^{cond}) in the coacervate phase from the intersection of the tie-line (purple) with the isorefractive line (red). The tie-line is determined by measuring the dilute-phase concentrations of ribozyme, c_r^{dil}, and peptide, c_p^{dil} (point A) and the average concentrations of ribozyme c_r^{tot} and peptide c_p^{tot} at which the system was prepared (point B). The isorefractive line depends on the refractive index difference Δn measured by QPI between a coacervate droplet and its surrounding phase and is calculated assuming a linear sum of contributions from ribozyme and peptide. B. Co-existing phases and tie-lines for each peptide system in the concentration plane on log scales. This is the same data as in Figure 4D in the main text, but now plotted against the molar concentration of the polymers rather than charge concentration. C. Same data as shown in B, now on linear scales. Error bars on coacervate composition represent error propagation via Jacobians (see Supplementary Information Section 5.3). For QPI measurements, N is provided in Table S10.

When considering the 7 tie-lines plotted in units of charge concentration (Figure 4), the tie-lines cluster into two groups on the basis of orientation in the composition plane. One group has steeper tie lines (slope near 1, group A) and a second group has a shallower tie-line (slope ~ 0.6, group B)). P-2, P-3, P-4, P-5, P-7 are in the group A, whilst P-1 and P-6 are in the group B.

Peptides with shallower tie-line slopes (group B) are enriched in polar non-charged residues, whilst those with tie-line slopes closer to 1 (group A) are depleted in these residues. Specifically, 3/5 of the non-charged residues in P-1 are polar (1xT + 2xS), and 3/6 of the non-charged residues in P-6 are polar (1xT + 2xS). In comparison, each member of group A has at most 1 non-charged residue that is

polar. This suggests that the number (or fraction) of polar non-charged residues could influence the tie-line slope in the composition plane.

The physical origin for this is not 100 % clear, but one possibility is that an increase in the number of polar non-charged residues shifts the equilibrium to favour coacervates which contain more water and ions and less polymer. Consistent with this, we observe a small decrease in the polymer volume fraction (Figure 4F) for P-1 of group B compared to group A coacervates (P-2, P-4) with the same charge. To test whether the fraction of polar non-charged residues is the dominant factor here, one could measure the phase behaviour for peptide sequences that have the same net charge but a varying number of polar residues.

On the basis of the tie-line length, the systems cluster into three well-separated groups: short (P-3, P-5, P-6), medium (P-1, P-2, P-4), and long (P-7). The distances between clusters is large compared to the spread within a cluster. The tie-line length could be due to the net charge where increasing the charge increased the length of the tie-line. The peptides with net charge +7 have short tie-lines and those with +8 all have medium tie-lines. The single peptide with charge + 13 has the longest tie-line.

Consistent with this, we note that similar tie-line lengths are obtained for peptides with a given net charge, whether the sequence includes charged residues with all of the same sign or some of the opposite sign (c.f. P-6 vs. P-3 and P-5). This also correlates with the theoretical expectation (and empirical precedent) in the literature on polyelectrolyte-complex coacervation[4] of oppositely-charged homopolymers that the tie-line length increases with the number of charges per chain.

5.5. Supplementary Information Note 4: Invariance of phase coexistence and increase in droplet size along tie-lines

To demonstrate that our procedure for preparing systems along a measured tie-line does indeed produce coacervate droplets of modulated size but comparable composition, we prepared P-6/HH_{min} samples at different concentrations of peptide (3 mM, 4 mM and 5 mM) and HH_{min} (1.7 mM, 2.3 mM and 2.9 mM) respectively, (shown in Figure S15A as points a, b, and c). These average compositions were chosen to lie on the tie-line that was determined for P-6 coacervates as described previously (Table S8). We next used QPI to measure the refractive index difference, Δn, of coacervate droplets prepared at points a, b, and c. Analysis of at least 900 droplets for each sample reveals that the distribution of Δn is comparable between samples (Figure S15B, red). This indicates that the concentrations in the coacervate are comparable across the samples prepared at different concentrations on the same tie-line (Figure S13). In contrast, P-6/HH_{min} coacervates prepared at a 2:1 charge ratio and offset from the determined tie-line (4 mM peptide: 2 mM HH_{min}, point d in Figure S15A) show a shift in the refractive index distribution relative to those prepared at points a, b, and c (Figure S15B, blue). This confirms that the latter sample (Point d) was prepared on a different tie-line compared to the other three samples, consistent with our expectation (Figure S15A). Taken together, these measurements demonstrate that our procedure to measure tie-lines is sufficiently accurate to predict tie-line location at concentrations at least 10-fold farther into the two-phase regime.

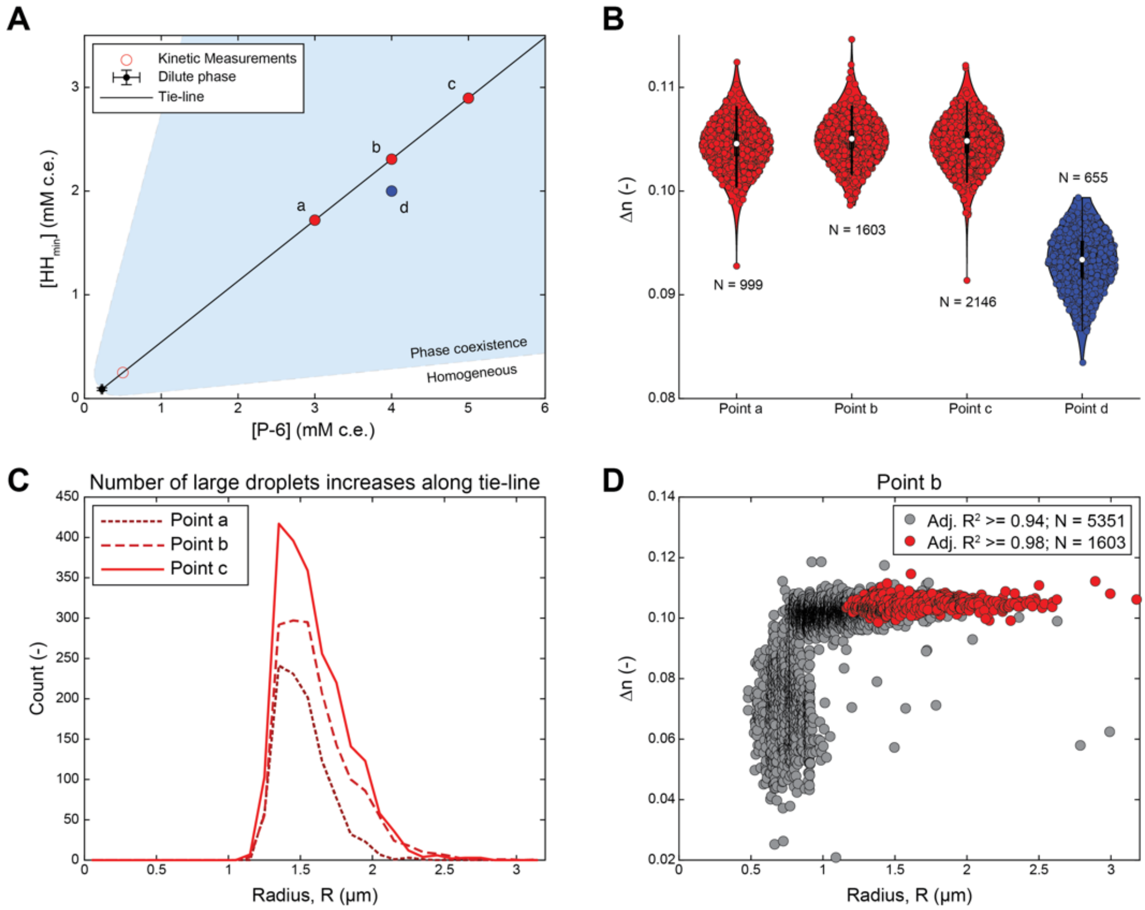

Figure S15: Invariance of phase coexistence along tie-lines. A. Schematic of phase diagram for the P-6/HH$_{min}$ system at low concentrations. Points a, b, and c (red) denote the average composition of samples prepared along the measured tie-line (black) passing through the composition point used for kinetic measurements (500 µM peptide, 250 µM HH$_{min}$; red circle). Point d (blue) represents a sample prepared at the same 2:1 charge ratio used in the kinetic measurements but at 4 mM peptide and lies off the previous tie-line. Note that the boundary between regions of homogeneous solution and phase coexistence is an approximation and provided as a visual aid. B. Refractive index distributions measured by QPI for samples prepared at points a, b, c, and d. The distributions contain measurements from N = 999, 1603, 2146 and 655 individual droplets, each fitted with an Adj. $R^2 \geq 0.98$. White circles denote medians and thick black bars show the interquartile range. Whiskers extend 1.5x beyond the interquartile range. The distributions measured for these three points are very similar, as expected for points on the same tie-line. As this tie-line was constructed to pass through the conditions used for kinetic measurements (red circle in A), the composition of coacervates formed at points a, b, and c are all equivalent to those formed at 0.5 mM peptide and 0.25 mM HH$_{min}$. In contrast, the refractive index distribution measured at point d deviates significantly from the others, confirming that point d is on a separate tie-line and corresponds to a different pair of coexisting phases. C. Droplet size distribution measured by QPI for samples prepared at points a, b, and c; Adj. $R^2 \geq 0.98$. The number of large droplets increases with increasing concentration along the tie-line. D. The refractive index difference measured for individual coacervate droplets from a sample prepared at point b. For droplets with a high fit-quality (Adj. $R^2 \geq 0.98$, red), Δn shows a small spread and is independent of droplet size, as expected for droplets of equivalent composition. Notably, all droplets with high fit-quality are relatively large (R > 1 µm). Using a lower threshold on fit-quality (Adj. $R^2 \geq 0.94$, grey) reveals that smaller droplets show a much larger spread. Preparing samples for QPI at higher concentrations (i.e. 4 mM peptide versus 0.5 mM) and on the measured tie-line ensures that the coacervate composition is equivalent with a sufficient number of large droplets for robust measurement with QPI.

From basic theoretical considerations, we expect the volume of coacervate phase to increase as the average composition moves along a tie-line and away from the dilute binodal (Figure S13). We hypothesized that this increase in total volume of coacervate phase would be accompanied by an

increase in the typical droplet size. To check this, we examined the size distribution obtained through our QPI experiments for P-6/HH_{min} coacervates prepared at points a, b, and c (Figure S15C). Consistent with our expectation, we find that the number of larger droplets increases as the average composition increases along a fixed tie-line. Note that only coacervates with a high-quality fit (Adj. R^2 >= 0.98) in the QPI analysis were included in these distributions. As we show below, this requirement excludes many smaller droplets. While the size distributions in Figure S15C therefore do not capture all droplets in the samples, they do suffice to demonstrate the increased numbers of droplets at larger sizes for samples prepared deeper in the two-phase regime.

To illustrate the influence of droplet size on QPI analysis, we examined the refractive index measured by QPI as a function of droplet size for P-6/HH_{min} samples prepared with 4 mM peptide and 2.3 mM HH_{min} (Figure S15D). These data are colored according to two thresholds for fit quality, with Adj. $R^2 \geq 0.94$ in grey and Adj. $R^2 \geq 0.98$ in red. For droplets with a high fit-quality, Δn shows a small spread and is independent of droplet size, as expected for droplets of equivalent composition. Notably, all droplets with high fit-quality are relatively large (R > 1 µm). Using a lower threshold on fit-quality reveals that smaller droplets show a much larger spread in Δn estimate. For coacervates smaller than ~ 1 µm in radius, the assumptions made in our image analysis pipeline are not fulfilled, yielding poor fit quality and a wide range of refractive index estimates. We expect that this large variation at small sizes comes from optical effects, such as Mie scattering, that feature prominently in this size range and are neglected in our analysis.

For the peptide/HH_{min} coacervates studied in our kinetic assays (500 µM peptide: 250 µM RNA), we find that droplets are typically too small for robust analysis by QPI when prepared at the conditions used for kinetic measurements. Preparing samples for QPI at higher concentrations (i.e. 4 mM peptide instead of 0.5 mM) and on the measured tie-line ensures that the coacervate composition is equivalent to that present in samples prepared at lower concentrations and that a suitable number of droplets are large enough for measurement. Taken together, our results show that preparing samples at higher concentrations along the same tie-line provides droplets of equivalent refractive index difference and larger size that enable more accurate analysis of QPI data.

5.6. Quantitative phase imaging and analysis

Quantitative phase imaging (QPI) was undertaken to determine the refractive index difference between the coacervate and its outer phase [6]. In brief, most QPI was performed with a Generation 2 coherence-controlled holographic microscope (Q-Phase, Telight (formerly TESCAN), Brno, CZ) based on Ref. [7] and equipped with an LED light source, though some data were acquired on an earlier Generation 1 Q-Phase system with a tungsten-halogen light source. In both cases, the light was filtered at λ = 650 nm with a 10-nm bandwidth notch filter. Samples were prepared as previously described by mixing RNA and peptide at a given concentration with 10 mM Tris, 1 mM $MgCl_2$ at pH 8.1. To increase the accuracy of the measurement, we increased the average droplet size by preparing samples at higher total concentrations (Table S9). To ensure that these larger droplets had the same thermodynamic properties as those used for our previous experiments, we used the measured tie-lines to determine the concentration of HH_{min} required for each system to remain on the same tie-line when

prepared with 4 mM of each peptide (see Supplementary Information Note 4). The coacervate droplets imaged with QPI were therefore prepared according to the concentrations provided in Table S9.

Table S9: Concentrations of RNA and peptide used for quantitative phase imaging

Peptide	[Peptide] (μM)	[HH_{min}] (μM)
P-1	4000	2426.7
P-2	4000	3213.2
P-3	4000	3397.4
P-4	4000	3414.4
P-5	4000	3358.7
P-6	4000	2307.5
P-7	4000	3337.0

Immediately after preparation, 5 μL of sample was loaded into a temperature-controlled flow cell. The flow cell was prepared from PEGylated coverslips (30 x 24 x 0.17 mm^3) adhered by heat to a sapphire slide (75 x 25 x 1 mm^3) with parafilm strips. The ends of the channel were sealed with the two-component silicone glue Twinsil (Picodent, Wipperfürth, DE), and the droplets were allowed to settle for ~ 10 minutes prior to measurement. The temperature of the slide was maintained at 25 °C for all measurements using water-cooled peltier elements as previously described[8].

Samples were imaged with a 40x dry objective (0.9 NA, Nikon) and the condenser set to an NA of 0.30. Typically, hologram z-stacks (Δz = 0.2 μm) were acquired for several fields of view with the first plane taken close to the surface of the cover glass. SophiQ v.9.2.415 software (Telight, Brno, CZ) was used to construct amplitude and compensated phase images from the raw holograms.

All phase images were subsequently analyzed using custom code written in MATLAB 2021b[6]. Briefly, individual droplets were identified in each image by intensity-based segmentation. For each identified droplet, Δn and the geometric parameters R, x_c, y_c, and Z_{eq} denoting droplet radius, (x,y)-coordinates of centroid, and height of the droplet's equatorial plane above the coverslip, respectively, were determined by fitting the measured phase shift $\Delta\varphi$ within a region of interest centered on the object to

$$\Delta\varphi(x,y) = \frac{2\pi}{\lambda}\Delta n H_{cap}(x,y|R,x_c,y_c,Z_{eq}) + \varphi_0 + A(Z_{eq},R). \quad (5.6)$$

Here, H_{cap} is the local thickness of a spherical cap and is given by

$$H_{cap}(x,y|R,x_c,y_c,Z_{eq}) = \sqrt{R^2-(x-x_c)^2-(y-y_c)^2}\left(1+\Theta\left(Z_{eq}^2+(x-x_c)^2+(y-y_c)^2-R^2\right)\right)\Theta(R^2-(x-x_c)^2-(y-y_c)^2) + Z_{eq}\Theta\left(R^2-Z_{eq}^2-(x-x_c)^2-(y-y_c)^2\right) \quad (5.7)$$

where $\Theta(x)$ is the Heaviside function. The same value of phase offset φ_0 is used for all droplets in an image, and set according to the Gaussian center of the image's pixel intensity histogram.

Finally, the third term $A(Z_{eq},R) = A_0(Z_{eq}-R)^2\Theta(Z_{eq}-R)$ is a regularisation function with $A_0 = 10^5$. Track.m (https://site.physics.georgetown.edu/matlab/index.html) was used subsequently to track all droplets within a z-stack through z. For each droplet, representative parameters are taken as those for which the Adj. R^2 from the fit was largest. All detected objects for which the best fit had Adj. R^2 < 0.98

were discarded. In each case, we found the Δn of individual droplets to be tightly and symmetrically distributed around a central mean, with coefficients of variation less than 2% (Figure S16, Table S10). Central statistics associated with the measured Δn distributions are presented in Table S10 below.

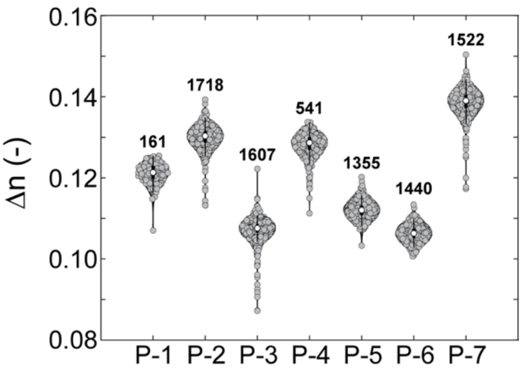

Figure S16: Variation in refractive index difference between coacervate systems. Distribution of refractive index differences (Δn) between individual coacervates (HH_{min}/peptide) and the surrounding dilute phase, as measured for each system by QPI. This data was used to determine the dense-phase concentrations reported in Figure 4. The number of individual coacervate droplets included in each measurement is given above the violin plots. White circles denote medians and thick black bars show the interquartile range. Whiskers extend 1.5x beyond the interquartile range.

Table S10: Statistics related to QPI measurements of coacervate refractive index

Peptide	Mean Δn (-)	Standard deviation (-)	Number of droplets analysed
P-1	0.12115	0.00236	161
P-2	0.13007	0.00212	1718
P-3	0.10741	0.00191	1607
P-4	0.12822	0.00247	541
P-5	0.11201	0.00151	1355
P-6	0.10633	0.00143	1440
P-7	0.13878	0.00239	1522

5.6.1. Determination of refractive index increments and partial specific volumes

The experimentally measured refractive index increment of 0.1655 ± 0.0045 ml/g for poly(A) RNA[6] was used as an estimate for the refractive index increment of HH_{min}. This was converted to units of $(\mu M)^{-1}$ by multiplication by $M_w \times \left(10^{-3}\,\frac{L}{mL}\right) \times \left(10^{-6}\,\frac{mol}{\mu mol}\right)/q_{net}$, where M_w and q_{net} are the sequence-dependent molar mass and net charge per molecule. A sequence-dependent estimate of the partial specific volume of HH_{min} was obtained by

$$\bar{\upsilon}_r = N_A \frac{\sum_j V_j}{\sum_j M_j}, \tag{5.8}$$

where V_j and M_j are the tabulated volume and molar mass of the jth ribonucleobase[9], and N_A is Avogadro's number. The refractive index increment and partial specific volume for HH_{min} are given in Table S11.

Table S11: Refractive index increment and partial specific volume of HH_{min}

RNA	$\frac{dn}{dc_r}$ (μM)$^{-1}$	Estimated Uncertainty (μM)$^{-1}$	$\bar{v}_r$ (ml/mg)
HH_{min}	5.436E-08	1.49E-09	0.5697

The refractive index increment for each peptide sequence were computed using the calculator tool within the SEDFIT software[10]. Here we briefly summarize the approach, which is detailed in[10]. The Wiener equation gives the refractive index increment $\frac{dn}{dc_p}$ of species p in solution as

$$\frac{dn}{dc_p} = \frac{3}{2}\bar{v}_p n_0 \frac{n_p^2 - n_0^2}{n_p^2 + 2n_0^2}, \tag{5.9}$$

where $\bar{v}_p$ is the partial specific volume of the solute p, n_0 is the refractive index of pure solvent, and n_p is the refractive index of the pure solute. The refractive index of each peptide is determined according to

$$n_p = \sqrt{\frac{2R_p + \bar{v}_p}{\bar{v}_p - R_p}}, \tag{5.10}$$

where the refraction per gram of peptide, R_p, is determined as a weighted-average over the sequence by

$$R_p = \frac{\sum_i R_i M_i}{\sum_i M_i}, \tag{5.11}$$

where R_i and M_i are the tabulated refraction per gram and the residue molar mass of the ith amino acid, respectively. The partial specific volume for each peptide is similarly determined by a weighted average over the sequence of the tabulated partial specific volumes for each amino acid $\bar{v}_i$ by

$$\bar{v}_p = \frac{\sum_i \bar{v}_i M_i}{\sum_i M_i}. \tag{5.12}$$

The refractive index increments and partial specific volumes for each peptide are given in Table S12 below. Note that, as with the RNA, the refractive index increments for each peptide were converted from units of L/g (output of Equation 5.9) to (μM)$^{-1}$ (listed in Table S12) through multiplication by $M_w \times \left(10^{-3}\frac{L}{mL}\right) \times \left(10^{-6}\frac{mol}{\mu mol}\right)/q_{net}$. For both the HH_{min} and the peptides, uncertainties in the increment values were estimated by rescaling the uncertainty in the experimental $\frac{dn}{dc}$ determination for polyA RNA from[6] according to

$$\delta\left(\frac{dn}{dc_i}\right) = \delta\left(\frac{dn}{dc_{polyA}}\right)\left(\frac{dn}{dc_i}/\frac{dn}{dc_{polyA}}\right). \tag{5.13}$$

Table S12: Refractive index increment and partial specific volume of peptides

Peptide	$\frac{dn}{dc_p}$ (μM)$^{-1}$	Estimated uncertainty	$\overline{v}_p$ (ml/g)
P-1	3.524E-08	9.7E-10	0.7350
P-2	3.942E-08	1.08E-09	0.7352
P-3	4.470E-08	1.23E-09	0.7616
P-4	3.772E-08	1.03E-09	0.7652
P-5	4.549E-08	1.25E-09	0.7647
P-6	4.144E-08	1.14E-09	0.7612
P-7	2.767E-08	7.6E-10	0.7424

5.7. Calculation of partition coefficients

The partition coefficient, *K*, is an equilibrium quantity defined as the ratio between the concentration of a species in one phase to another. In this work, the partitioning is defined relative to the dilute phase, such that the partition coefficient of the ith species is given by

$$K_i = \frac{c_i^{cond}}{c_i^{dil}}, \tag{5.14}$$

where c_i^{cond} and c_i^{dil} are the concentrations of the *i*th species in the condensed (coacervate) and dilute phases, respectively. Partition coefficients for HH_{min} and peptide are given for each peptide/HH_{min} system in Figure S17.

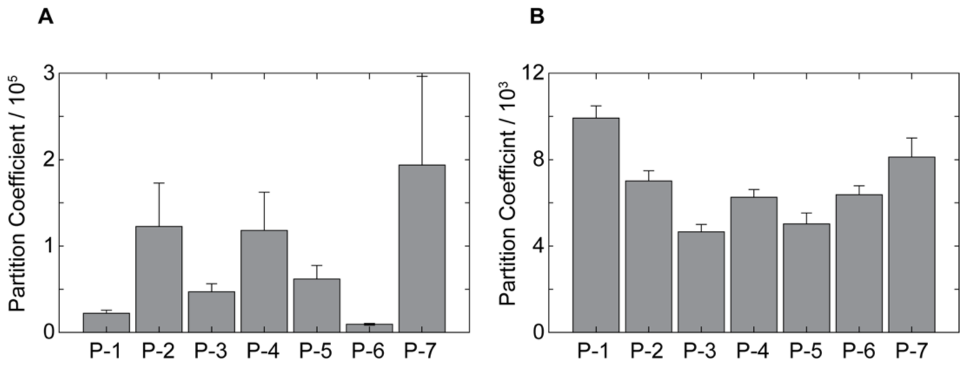

Figure S17: Ribozyme and peptide partition coefficients. A. Partition coefficient of HH_{min} for each HH_{min}/peptide system, calculated as the ratio of concentrations of HH_{min} in the coacervate phase relative to the dilute phase. B. Partition coefficient of peptide for each HH_{min}/peptide system, calculated as the ratio of concentrations of peptide in the coacervate phase relative to the dilute phase. Error bars represent an estimate of the uncertainty and were obtained by propagating the uncertainty in the concentration measurements from each phase assuming no correlations.

5.8. Calculation of the coacervate-phase volume fraction and polymer volume fraction in the coacervate phase

In this work, we distinguish between two different types of volume fractions, which we term the phase volume fraction and the species volume fraction in a given phase. The phase volume fraction is a measure of how much of that phase is present in the system (Figure S13A, B). For a system composed of multiple coexisting phases, the phase volume fraction of phase α is given by

$$\phi_\alpha = \frac{V_\alpha}{V_{tot}}, \tag{5.15}$$

where V_{tot} is the total volume of the system and V_α is the volume of phase α. If phase α is present in the system as multiple disconnected droplets, then V_α is the sum of the volumes of all of these droplets. Direct measurement of this volume experimentally is prone to underestimation, as incidental wetting to sample boundaries leads to droplets that are not included in the total. To avoid these issues, we determined the coacervate-phase volume fraction instead using the lever rule

$$\phi_\alpha = \frac{c^{tot} - c^{dil}}{c^{cond} - c^{dil}}. \tag{5.16}$$

Here, the denominator is a measure of the total length of the tie-line (distance between binodal branches) and the numerator gives the distance along the tie-line from the dilute-branch to the total average composition of the system. Note that any species can be used to calculate the phase volume fraction via the lever rule. Dense-phase volume fractions corresponding to the QPI experiments are given for each peptide/HH_{min} system in Figure S18A.

By contrast, the species volume fraction in a phase is a measure of the amount of a particular species in a given phase, and is defined as:

$$\phi_i^\alpha = c_i^\alpha \bar{v}_i \tag{5.17}$$

where c_i^α is the concentration of the ith species in phase α. Note that we assume here that the partial specific volumes are independent of the phase. Finally, we use the polymer volume fraction in the condensed phase to denote the sum of the peptide and HH_{min} volume fractions in the condensed phase

$$\phi_{polymer}^{cond} = c_p^{cond} \bar{v}_p + c_r^{cond} \bar{v}_r. \tag{5.18}$$

Volume fractions of ribozyme and peptide in the dense phase are given for each peptide/HHmin system in Figure S18B.

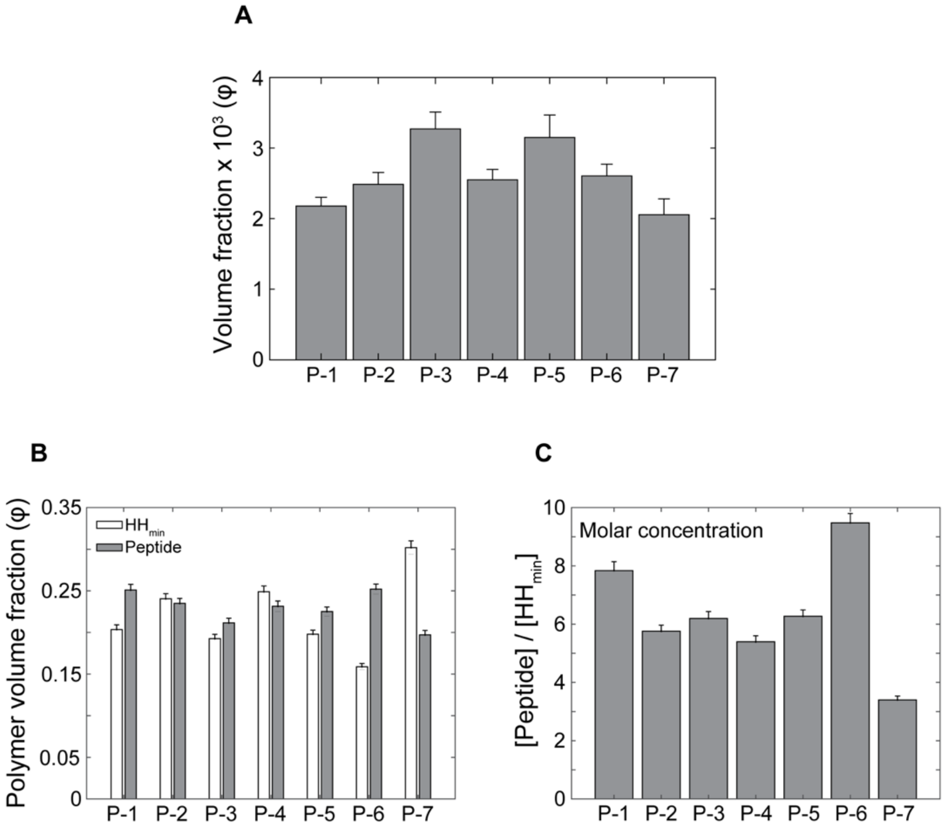

Figure S18: Comparison of coacervate volume fraction and coacervate composition between systems. A. Amount of condensed phase for each system, given as a fraction of the total sample volume calculated by the lever rule using the average concentrations given in Table S9. B. The fraction of the condensed-phase volume occupied by ribozyme and peptide, respectively, for each system. The sum of the peptide and ribozyme volume fractions is given in Figure 4F in the main text. C. The molecular stoichiometry in the condensed phase, calculated for each system as the ratio of peptide concentration in the condensed phase to ribozyme concentration in the condensed phase, with both given as moles of polymer per unit volume. Error bars in A and C represent an estimate of the uncertainty and were obtained by propagating uncertainties in each of the relevant concentration measurements assuming no correlations. Errorbars in B represent the uncertainties for the dense-phase concentrations in Figure 4D rescaled by the partial specific volume of the corresponding polymer using values listed in Tables S11 and S12.

6.0. Fluorescence recovery after photobleaching: ribozyme diffusion inside droplet

FRAP data was background subtracted, normalized and plotted using GraphPad Prism 9.4 software. The diffusion coefficient was then calculated using the following formula:

$$D = 0.224 \frac{r_n^2}{t_{1/2}} \ \mu m^2/s \qquad (6.1)$$

where r_n is the radius of the bleached area, $t_{1/2}$ is the half-time of recovery and the 0.224 numerically determined value given in the literature[11, 12]. Recovery profiles and representative images for each peptide/HH_{min} system are shown in Figure S19.

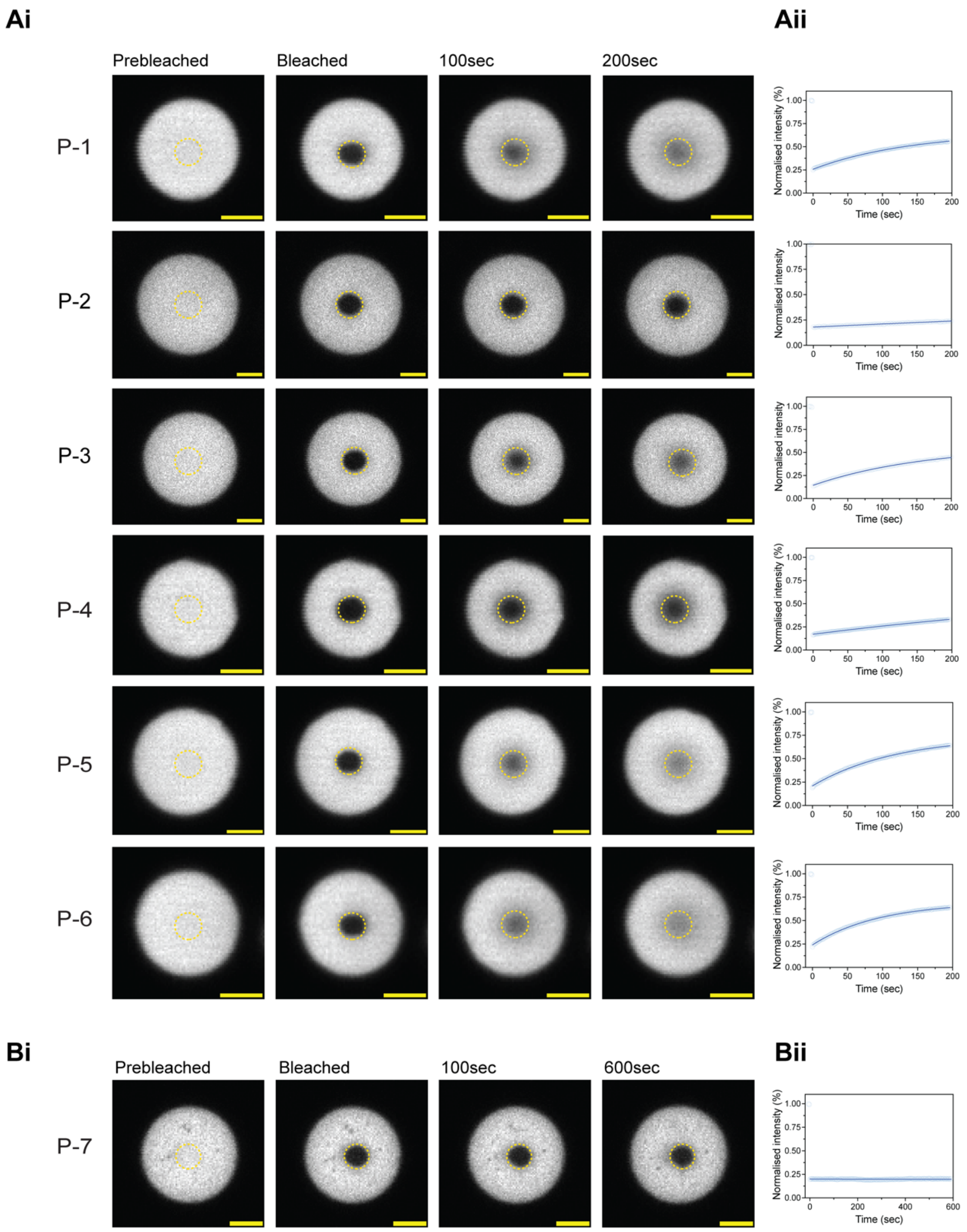

Figure S19: HH_{min} diffusion measurement using FRAP inside RNA/peptide coacervate systems. Ai. Confocal microscopy images show prebleached, bleached, and FRAP recovery after 100 and 200 s. Peptide numbers marked left of the droplet images indicate HH_{min} partner peptide. Aii. The recovery curve of the bleached area with respect to time; circles denote data and solid line shows the fit to the data. Fluorescence intensity was normalised with maximum values of each set. Bi. HH_{min} single droplet FRAP with P-7 peptide. Images show negligible FRAP recovery till 600 s. Bii. The plot of the FRAP recovery of the bleached region over time shows no recovery. Circles show data obtained from imaging; solid line is a fit to the data. Scale bar 5 μm. Images are representative of more than three experiments.

7.0. RNase A footprinting assay

To determine an appropriate nuclease concentration for the RNase A cleavage reaction, 25 μM FAM-HH (in the absence of peptides) was treated with RNase A prepared in 100 nM, 10 nM and 1 nM concentrations in 10 μL of reaction buffer in a centrifuge tube and incubated for 8 min at room

temperature. 100 nM RNaseA concentration was chosen for the experiment, which showed optimum band intensities of cleaved RNA fragments (Figure S20A). HH_{min} ladder was prepared with 20 µM (molar concentration) FAM-HH, to which yeast RNA (from RNase kit, Thermo Fisher) and alkaline hydrolysis buffer (from RNase kit) was added and incubated at 95 °C for 15 min and after resting inside ice for 15-20 min transferred to -20 °C freezer.

For the peptide/RNA interaction analysis 25 µM FAM-HH and 50 µM peptide were mixed in 10 µL of buffer in centrifuge tubes and allowed to settle for 15 min. After addition of 100 nM RNase A, reaction mixtures were incubated for 12-15 min at room temperature. To quench the reaction, 20 µL of 2x RNA loading dye mixed with 20 mM sodium hexametaphosphate was added to the solution and immediately transferred to -20 °C in a freezer for 15 min. The samples (2 µL for untreated HH_{min} and 4 µL for all treated HH_{min}) were run on denaturing 20 % urea-PAGE and imaged using a Typhoon 9500 Fluo Phospho Imager with the 473 nm laser with the filter model -BPB1/530F-20 (bandwidth 520-540 nm). Band intensities were quantified using GraphPad Prism 9.4. For each lane, the percent relative band intensity obtained by

$$\frac{Band\ fluorescence\ intensity}{Total\ sum\ of\ all\ band\ fluorescence\ intensity} \times 100 \qquad (7.1)$$

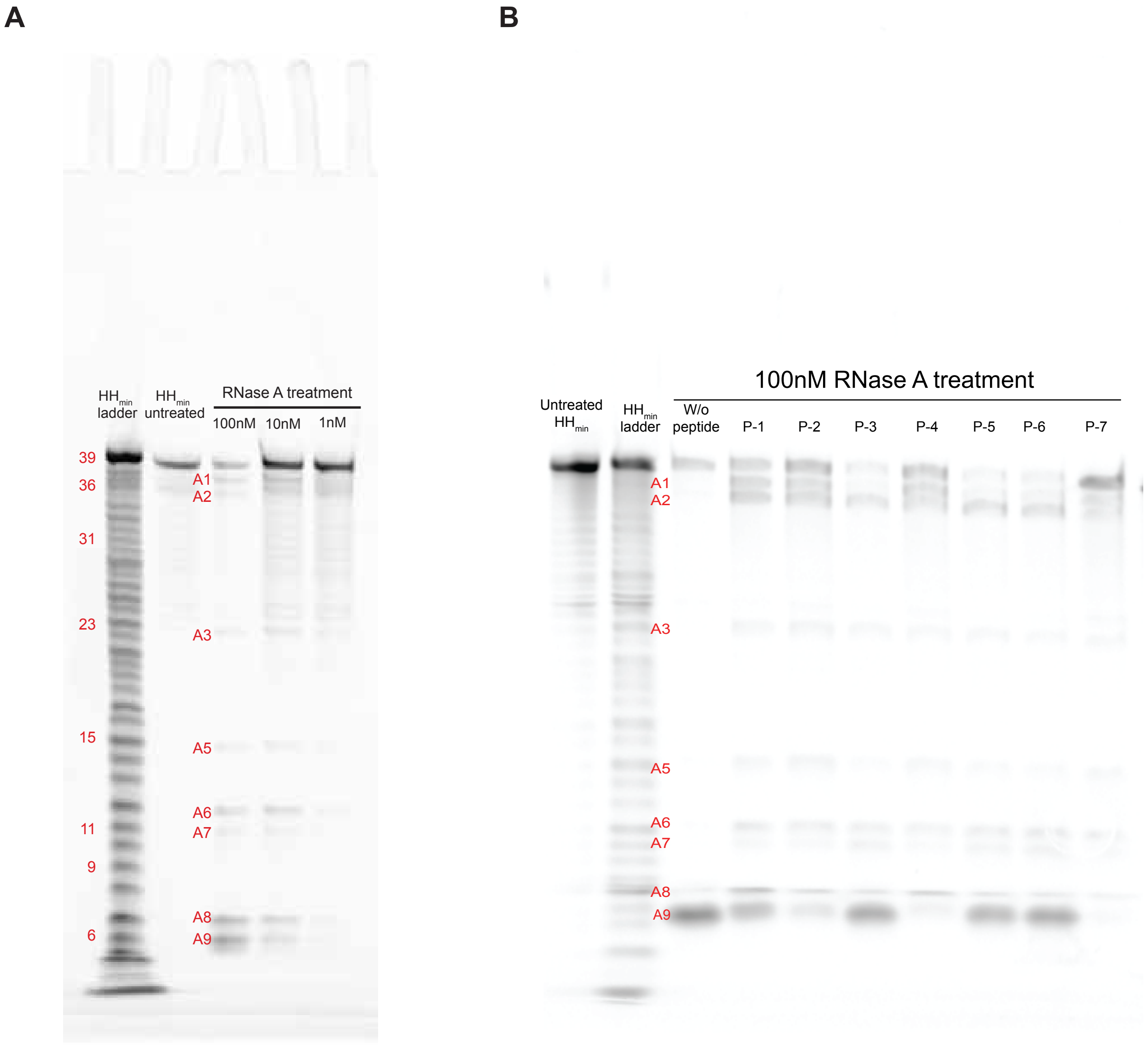

Figure S20: Results from the RNase A footprinting assay. A. Optimisation of RNase A concentration. Gel image showing band intensities of RNase A cleavage sites on HH_{min}. The lanes are presented as follows: HH_{min} ladder generated by alkaline hydrolysis of HH_{min} with individual degraded nucleotides (37 visible out of 39); untreated HH_{min}; HH_{min} treated with 100 nM, 10 nM and 1 nM RNase A. We note that there is no band intensity at A4; it is possible that this site was uncleavable because of the folded region of the ribozyme. B. RNase A treatment of HH_{min} with and without peptide. Gel image showing band intensities of RNase A cleavage sites on HH_{min}. Lanes show untreated HH_{min}; a ladder generated by alkaline hydrolysis of HH_{min} with individual degraded nucleotides (37 visible out of 39); HH_{min} with and without peptide following 100 nM RNase A treatment for 12 min. HH_{min} charge concentration was 25 μM, peptide charge concentration was 50 μM. This is an experimental replicate of Figure 6B and includes the control experiment of RNase A cleavage of HH_{min} without peptide. Uncropped gels are provided in the data repository.

8.0. Supplementary Information Note 5: Determination of pH within the coacervate droplet.

8.1. Motivation and model

Given that ribozyme activity is linearly dependent on the pH[13, 14], we sought to determine if differences in pH could explain the 15-fold difference in the rate we observed between the P2 and P3

coacervate systems. The literature indicates that a 15-fold difference in ribozyme rate corresponds to a difference of 1.5 pH units[13]. We chose to use a theoretical approach to estimate the pH of the dense phase of the peptide/HH_{min} coacervate due to experimental challenges that arise from direct measurement of small volume fractions of the condensed phase. The volume fraction of the condensed phase is typically $\phi^{II} \approx 2 \times 10^{-4}$ (Table S13), such that a 1 mL sample contains only ~0.2 µL of condensed phase. In addition, pH-probes can have different fluorescent properties in a macromolecularly-dense coacervate phase (with solvent volume fraction ϕ_s between 0.5 and 0.6) compared to dilute aqueous solutions. This makes it challenging to obtain the true pH in the condensed phase from calibrations obtained in the dilute phase (with $\phi_s \cong 1$). In the theoretical approach developed here, we assume that the proton number (N^{tot}) is conserved upon phase separation to estimate the pH in the dense phase (pH^{II}) from the pH measured in the dilute phase (pH^{I}) and the starting pH of the coacervate components (pH^{tot}), i.e. the pH in the RNA HH_{min} and peptide solutions prior to mixing.

Table S13: Condensed-phase volume fraction[a]

System	ϕ^{II}
P-1/HH_{min}	1.842 x 10^{-4}
P-2/HH_{min}	1.858 x 10^{-4}
P-3/HH_{min}	2.209 x 10^{-4}
P-4/HH_{min}	1.789 x 10^{-4}
P-5/HH_{min}	2.195 x 10^{-4}
P-6/HH_{min}	1.883 x 10^{-4}
P-7/HH_{min}	1.493 x 10^{-4}

[a] Condensed-phase volume fraction represents the fraction of the system volume occupied by the condensed phase and depends on the total concentrations added. Values presented here are for 500 µM peptide and 250 µM HH_{min}.

Prior to mixing, the total number of (hydrated) protons in solution (N^{tot}) is set by the pH of the Tris HCl buffer (8.1). We note that, since the peptides are added as a protonated chloride salt and the RNA as a deprotonated sodium salt, each with pK_a values far from the pH of the buffer, the presence of these polyelectrolytes in solution doesn't appreciably alter N^{tot}. After mixing the peptide with HH_{min} and subsequent phase separation, N^{tot} may change if there is a significant shift in the protonation equilibrium of a species in one or both of the resulting phases. For instance, this could result from a phase-specific shift in protonation pK_a of a species away from the standard dilute-solution value and/or a shift in the local pH within a phase away from pH^{tot} and towards a solute pK_a. Here we assume that there are no pK_a shifts and solve for the local pH in the limit that the values are sufficiently far from the pK_a values relevant for our peptides and RNA such that N^{tot} is unchanged by phase separation. As we shall see, the local pH values we obtain are self-consistent with this limit. Consequently, the number of (hydrated) protons in the dense phase is given by $N^{II} = N^{tot} - N^{I}$. Converting to molar proton concentration ($c = \frac{N}{V}$) and assuming no change in solution volume upon phase separation ($V^{tot} = V^{I} + V^{II}$), this becomes

$$c^{II} = \frac{1}{\phi^{II}} c^{tot} + \left(1 - \frac{1}{\phi^{II}}\right) c^{I}, \tag{8.1}$$

where $\phi^{II} = \frac{V^{II}}{V^{tot}}$. This equation relates the concentration of (hydrated) protons in the condensed phase to that in the dilute phase and the total concentration of protons prior to phase separation.

pH is conventionally defined in terms of the activity rather than concentration of (hydrated) protons: $pH \equiv -\log_{10}(a_{H^+})$; this is an important distinction for solutions with high ionic strength, like coacervates, in which electrostatic interactions cause ion behavior to deviate from that of ideal solutes. To calculate pH^{II} from Equation 8.1, the concentrations must therefore be rewritten in terms of activities. The activity of a species in phase α may be written in terms of the molal proton concentration in that phase (b^α) as $a^\alpha = \frac{\gamma^\alpha b^\alpha}{b^\circ}$, where γ^α is a phase-specific activity coefficient equal to 1 in the ideal limit and $b^\circ = 1$ mol/kg solvent is a phase-independent constant that defines the molality scale. The phase-specific molal concentration is related to the local molar concentration (c^α) by $b^\alpha = \frac{c^\alpha}{\rho_s \phi_s^\alpha}$, where $\rho_s \approx 1$ kg/L is the solvent mass density and ϕ_s^α is the solvent volume fraction in phase α.

Combining these relations together, the dense-phase pH is given by

$$pH^{II} = -\log_{10}\left[\frac{\gamma^{II}}{\phi_s^{II}}\right] - \log_{10}\left[\left(\frac{1}{\phi^{II}}\right)\left(\frac{\phi_s^{tot}}{\gamma^{tot}}\right)10^{-pH^{tot}} + \left(1 - \frac{1}{\phi^{II}}\right)\left(\frac{\phi_s^{I}}{\gamma^{I}}\right)10^{-pH^{I}}\right]. \quad (8.2)$$

This expression contains nine parameters, of which two (pH^I and pH^{tot}) are measured directly and two are inferred from measurements of condensed-phase composition (ϕ_s^{II}) and the phase diagram (ϕ^{II}). Since the (poly)electrolyte concentrations are small in the dilute phase and also when averaged over the entire system, we approximate $\phi_s^{tot} \cong \phi_s^I \cong 1$ and $\gamma^{tot} \cong \gamma^I \cong 1$. This leaves a single undetermined parameter, the dense-phase proton activity coefficient γ^{II}, from which the pH of the condensed phase can be determined.

Owing to the high charge concentrations in the dense phase, it is unreasonable to approximate protons there as ideal. We therefore applied Debye-Hückel theory to generate a rough numerical estimate for γ^{II}. The Debye-Hückel limiting law[15] is commonly used to estimate the mean activity coefficient applicable to a pair of electrolytes with charges z_+ and z_- in a solution of ionic strength I as $\log_{10}(\gamma_\pm) = -A|z_+ z_-|\sqrt{I}$. We take $z_+ = 1$ for the proton and select Cl^- as the anion such that $z_- = -1$. In the dense phase, the prefactor is given by

$$A^{II} = \frac{F^3}{4\pi N_A \ln(10)}\left(\frac{\rho_s \phi_s^{II} b^\circ}{2(\varepsilon^{II})^3 R^3 T^3}\right)^{1/2}, \quad (8.3)$$

where F is the Faraday constant, N_A is Avogadro's number, ε^{II} is the local dielectric constant, R is the gas constant and T is the temperature. We have included the factor ϕ_s^{II} to account for the reduced solvent volume in the dense phase compared to typical dilute solutions. We estimate the dense-phase dielectric constant as a weighted sum of contributions from solvent, peptide and nucleic acid as

$$\varepsilon^{II} \approx \varepsilon_0 \sum_i \varepsilon_i \phi_i^{II} \quad (8.4)$$

using measured species volume fractions in the dense phase (ϕ_i^{II}, Table S14) and literature values of the relative permittivities (ε_i) for viral protein[16], DNA[16], and water[23] (see Table S15). ε_0 is the static permittivity of free space. To estimate the activity coefficient for protons in the dense phase from the Debye-Hückel expression, we need to estimate the remaining parameter, the ionic strength in the dense phase.

Table S14: Molecular composition (volume fraction) within condensed phase[a]

System	ϕ_p^{II}	ϕ_r^{II}	ϕ_s^{II}
P-1/HH_{min}	0.2510	0.2036	0.5454
P-2/HH_{min}	0.2349	0.2404	0.5246
P-3/HH_{min}	0.2115	0.1926	0.5960
P-4/HH_{min}	0.2314	0.2490	0.5196
P-5/HH_{min}	0.2252	0.1979	0.5769
P-6/HH_{min}	0.2521	0.1589	0.5890
P-7/HH_{min}	0.1971	0.3020	0.5009

[a] Solvent volume fraction in condensed phase is calculated as $\phi_s^{II} = 1 - \phi_p^{II} - \phi_r^{II}$. Deviation of row sums from 1 represents rounding error.

Table S15: Approximate dielectric constants of primary coacervate molecular species

Species	Symbol	Value
peptide	ε_p	3[a]
HH_{min}	ε_r	8[b]
water	ε_s	78.3[c]

[a] Typical value for protein based on measurements of shell and tail proteins from T7 virus. Ref: [16]
[b] We use the value measured for packaged viral DNA (Ref: [16]) as a proxy for RNA.
[c] Measured value for water at 25 °C. Ref: [17].

While all charged species contribute to the ionic strength, we expect the high concentrations of peptide and HH_{min} polyelectrolytes in the dense phase to dominate. Accordingly, we make the simplifying approximation that contributions to the ionic strength from all other charged species are negligible. Ionic strength calculations for polyelectrolyte solutions are generally complex[18,19, 20], owing in large part to the many spatial correlations among charges, such as those from chain connectivity and ion pairing. However, the molar-scale charge-equivalent concentrations we measure in these coacervate systems (Figure 4) suggest that the length-scale for electrostatic screening is comparable to or even smaller than the other relevant correlation lengths. We therefore calculate the ionic strength in the limit that all spatial ion correlations (including those from chain connectivity) are negligible, which can be considered a mean-field limit.

In this limit, the equation for the dense-phase ionic strength takes the usual form for disconnected low-valence ions:

$$I^{II} = \frac{1}{2b^\circ} \sum_j b_j^{II} \left(z_j^{mon}\right)^2, \tag{8.5}$$

where the sum includes terms for peptide and HH_{min} only. b_j^{II} is the dense-phase molality of polyelectrolyte species j in units of charge equivalents and determined experimentally (Figure 4). z_j^{mon} is the punctate charge of a monomer along the backbone, and is +1 for cationic amino acid sidechains and -1 for phosphates along the RNA backbone. The proton activity coefficient γ^{II} is then given by the Debye-Hückel limiting law using Equations 8.3-8.5 above. In Table S16, we report the values of ε^{II}, I^{II}, and γ^{II} estimated from these equations. With γ^{II} in-hand, the dense-phase pH may be computed from Equation 8.2.

Table S16: Estimated dense-phase proton activity coefficients and associated parameters[a]

System	ε^{II}	I^{II}	γ^{II}
P-1/HH$_{min}$	45.09	2.617	0.0401
P-2/HH$_{min}$	43.71	2.688	0.0351
P-3/HH$_{min}$	48.84	1.835	0.0824
P-4/HH$_{min}$	43.37	2.713	0.0338
P-5/HH$_{min}$	47.43	1.961	0.0704
P-6/HH$_{min}$	48.15	1.959	0.0728
P-7/HH$_{min}$	42.23	3.458	0.0201

[a] All parameters are dimensionless.

8.2. Experimental determination of pH^I and pH^{tot}

Dilute-phase pH values were measured by direct placement of a calibrated micro pH meter electrode (Mettler Toledo) into the supernatant of a centrifuged coacervate dispersion. The measured pH^I values are 8.17 ± 0.02 for the P-2 system and 8.13 ± 0.02 for the P-3 system. In both cases, these values represent the mean ± standard deviation from 3 replicates prepared in parallel. For both systems, we take $pH^{tot} = pH^{buffer}$, where pH^{buffer} is the pH of the buffer used to prepare the solutions and which was measured to be 8.10 ± 0.02 (mean ± standard deviation of 3 replicates).

8.3. Generation of simulated pH^{II} data via Monte Carlo

Simulated distributions of internal pH presented in Figure S21 were generated from Equation 8.2 using the Monte Carlo method[21] to obtain values of pH^I and pH^{tot} from Gaussian distributions constrained by measurements (described in Section 8.2 above). In the Monte Carlo simulation, the mean and standard deviations of the Gaussian distributions were set to the measured central value and experimental uncertainty, respectively. Other parameter values were treated as constant and taken from Table S13 (ϕ^{II}), Table S14 (ϕ_s^{II}), Table S16 (γ^{II}), or taken as 1 for ϕ_s^{tot}, ϕ_s^I, γ^{tot}, and γ^I. For each run, $n_{draw} = 1 \times 10^7$ independent samples were drawn for each of pH^I and pH^{tot} and then randomly paired. Individual pairs for which pH^I was below the threshold

$$pH^{thresh} = pH^{tot} - \log_{10}\left[\left(\frac{\gamma^I}{\gamma^{tot}}\right)\left(\frac{\phi_s^{tot}}{\phi_s^I}\right)\left(\frac{1}{1-\phi^{II}}\right)\right] \quad (8.6)$$

were discarded, as they would correspond to the unphysical situation of negative proton numbers in the dense phase. Typically, more than 8.5×10^6 pairs remained. pH^{II} was then calculated from Equation 8.2 for each surviving pair to populate the distributions in Figure S21A. The distribution of ΔpH^{II} in Figure S21B was generated by pairing samples from the simulated pH^{II} distributions for the P-3 and P-2 systems and taking differences. To assess uncertainty in the most likely value of ΔpH^{II} as well as uncertainty in the probability that $\Delta pH^{II} \geq$ 0.5, 1.0, or 1.5, the entire process was replicated 100 times. Uncertainties reported for these quantities represent the standard deviation observed over the 100 replicates. From 100 independent simulated datasets, the most likely difference between the internal pH of P-3/HH$_{min}$ and P-2/HH$_{min}$ coacervates is -0.0301 ± 0.0006 (red vertical line). That said, significantly larger positive differences are observed with substantial probability. In particular, the probabilities that ΔpH^{II} is at least as large as 0.5, 1.0 or 1.5 are 0.1449 ± 0.0001, 0.03951 ± 0.00007,

and 0.01183 ± 0.00003. These results suggest a probability of less than 2% that the difference in the dense-phase pH between the P-2 and P-3 systems is large enough for the 15-fold reaction rate difference to be accounted for entirely by the pH-dependence of HH_{min}. Much more likely is that other factors, including additional aspects of the coacervate microenvironment, contribute significantly as well.

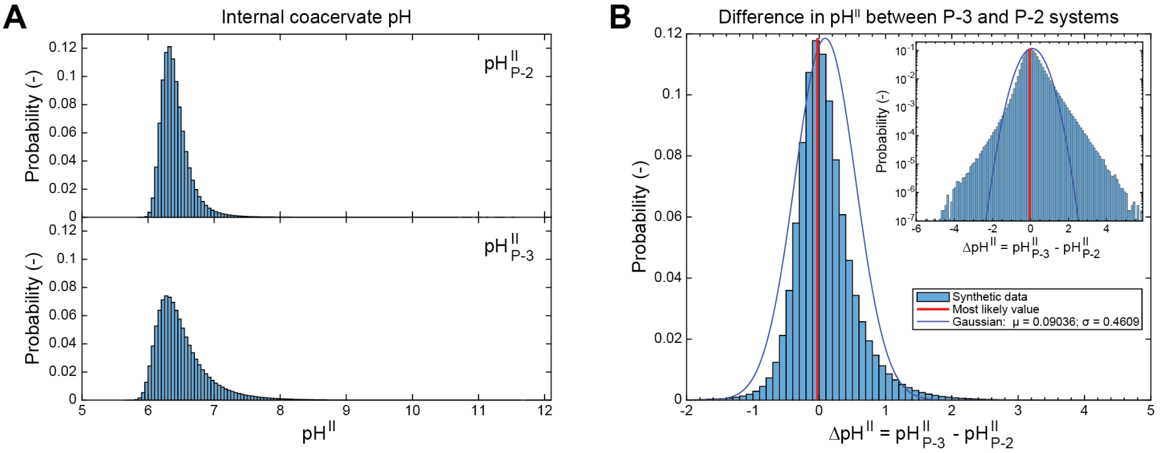

Figure S21: Simulated pH inside coacervates. A. Distribution of plausible condensed-phase (i.e. internal) pH values for the P-2/HH_{min} (top) and P-3/HH_{min} (bottom) systems. Distributions were calculated from Equation 8.2 using values of pH^{tot} and pH^{I} drawn via the Monte Carlo method from Gaussian distributions parameterized by the experimentally-measured central value and uncertainty of the buffer pH (8.10 ± 0.02) and dilute-phase pH (8.17 ± 0.02 and 8.13 ± 0.02 for P-2 and P-3, respectively). Values of other parameters are held constant. For the P-2/HH_{min} system: $\gamma^{II} = 0.0351$, $\phi_s^{II} = 0.5246$, and $\phi^{II} = 1.858 \times 10^{-4}$. For the P-3/$HH_{min}$ system: $\gamma^{II} = 0.0824$, $\phi_s^{II} = 0.5960$, and $\phi^{II} = 2.209 \times 10^{-4}$. For both systems, we take $\frac{\phi_s^{tot}}{\gamma^{tot}} \cong 1 \cong \frac{\phi_s^{I}}{\gamma^{I}}$. Histograms represent ~8.5 x 10^6 independent samples. Bin-width is 0.05 pH-units. The most probable values of the internal pH are near ~6.3 for both systems. The distributions are highly asymmetric with pronounced tails extending to pH 12 (though difficult to see on linear scales). B. Distribution of plausible differences in internal pH between the P-3/HH_{min} and P-2/HH_{min} systems: $\Delta pH^{II} = pH^{II}_{P-3} - pH^{II}_{P-2}$. The ΔpH^{II} distribution was populated from differences between internal pH values drawn from the distributions in A. The ΔpH^{II} distribution is peaked near zero and non-Gaussian, displaying instead a narrower peak and also more pronounced tails. A Gaussian distribution (blue line) with the same mean (μ) and standard deviation (σ) as the synthetic ΔpH^{II} distribution is shown for reference. The difference in the tails is most obvious when viewed on a semi-log plot (inset). Bin-width is 0.1 pH-units.

8.4. Self-consistency assessment of physical limits applied to pH-related calculations

We briefly check for self-consistency between our results and the two physical limits taken in the calculations. First, the dense-phase ionic strength I^{II} was estimated in the limit that the electrostatic screening length (Debye length) is smaller than or comparable to the length-scale associated with ion correlations from chain connectivity. To check this, we use the I^{II} estimates to calculate the corresponding Debye length in the dense phase as

$$\lambda_D = \sqrt{\frac{\varepsilon^{II} k_B T}{2 \rho_s \phi_s^{II} F^2 I^{II} b^{\circ}}} \tag{8.7}$$

and report the resulting values in Table S17. For each coacervate system, we find the screening length is $\lambda_D \leq 0.23$ nm. This is smaller than the typical distance between adjacent monomers for polypeptides (3.65 Å) [22] and also the phosphate-phosphate distance on nucleic acids (3.4 Å), consistent with the limit taken.

Table S17: Estimated Debye length in dense phase[a]

System	λ_D (nm)
P-1/HH_{min}	0.1933
P-2/HH_{min}	0.1914
P-3/HH_{min}	0.2298
P-4/HH_{min}	0.1907
P-5/HH_{min}	0.2227
P-6/HH_{min}	0.2221
P-7/HH_{min}	0.1698

[a] Calculated based on the ionic strengths given in Table S16.

The second important limit is that local pH values remain sufficiently far from polyelectrolyte pK_a values such that the total number of hydrated protons in solution does not change appreciably upon phase separation. To check this, we compare the local pH values for the P-2/HH_{min} and P-3/HH_{min} systems to tabulated pK_a values of the charge groups in the polyelectrolytes. We take the sidechain pK_a values of lysine and arginine to be 10.9 and 13.8[23], respectively, and the phosphate pK_a in the RNA backbone to be ~2. In all cases, these pK_a values are several pH units away from the pH measured in the dilute phase (8.17 and 8.12) and also from the most likely pH value in the dense phase obtained via simulation (~6.3 in both cases). These local pH values are thus far from the standard pK_a values of the polyelectrolyte charge groups, consistent with the limit taken.

Finally, we note that any errors introduced through the limits and approximations employed here are systematic rather than statistical and will impact estimates for each peptide/HH_{min} system similarly. In particular, systematic errors in γ^{II} will modulate the pH distributions in Figure S21 quantitatively. However, we expect the general distribution shapes and our conclusion that the most likely difference in internal pH between the P-3 and P-2 systems is $0 < |\Delta pH^{II}| \ll 1.5$ are both insensitive to correlated variation in γ^{II} from such systematic errors.

9.0 Uncropped gels from data presented in the main text and supplementary information

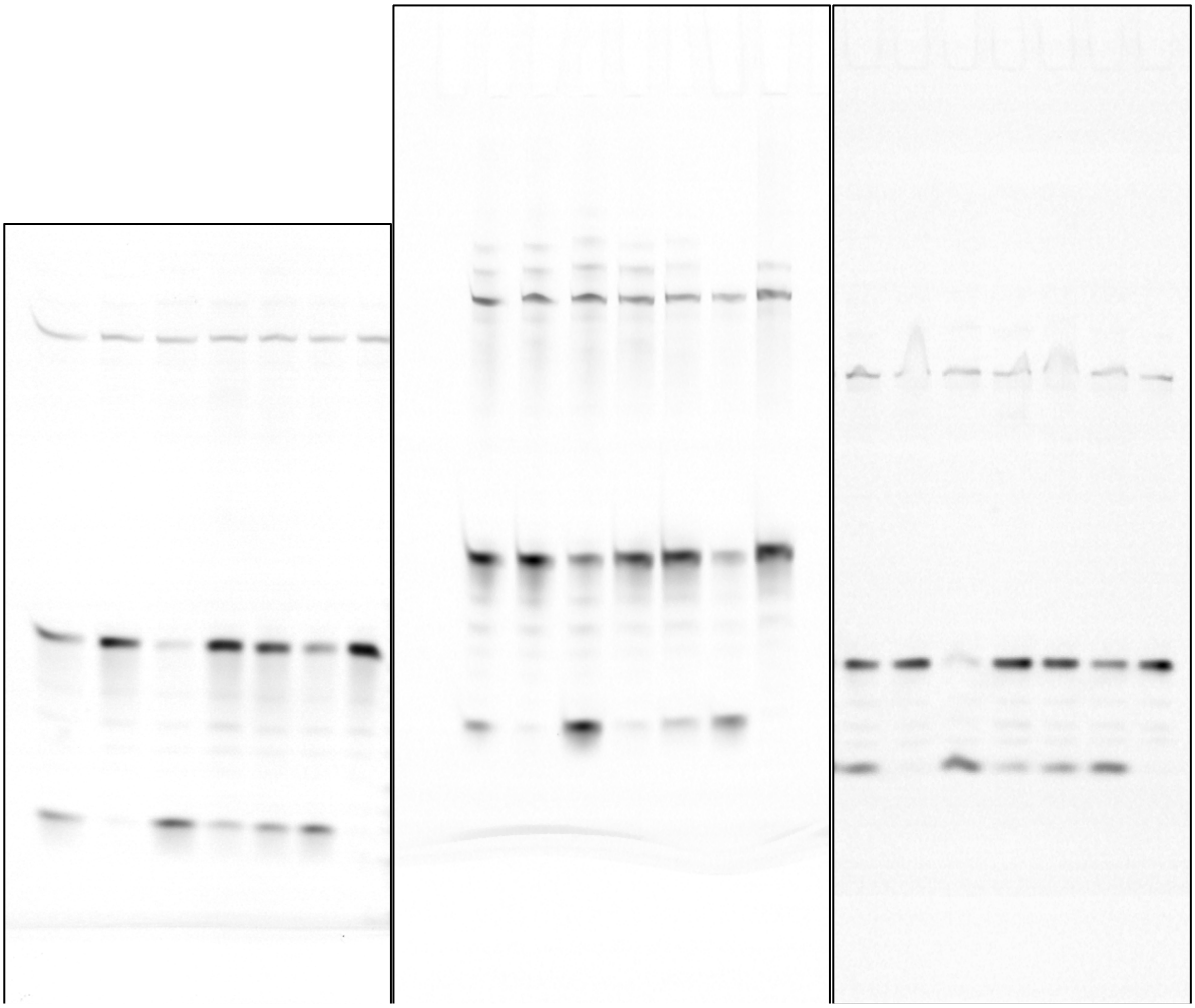

Figure S22: Gel from figure 2B (Left) and uncropped gels of replicates (middle and right).

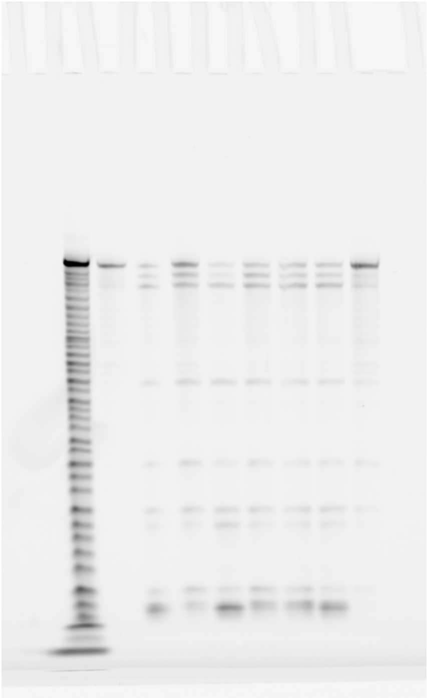

Figure S23: Uncropped gel from Figure 6B

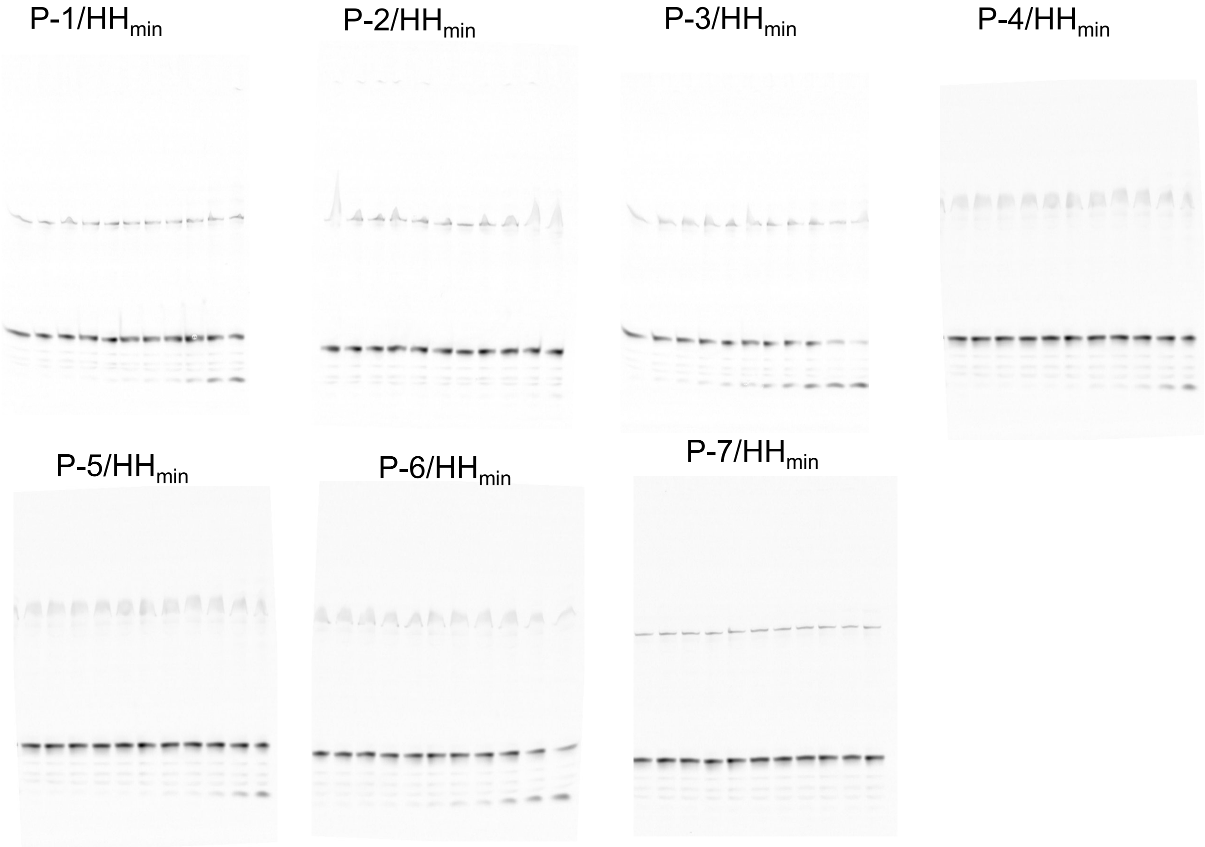

Figure S24: Uncropped gel image from Figure S3.

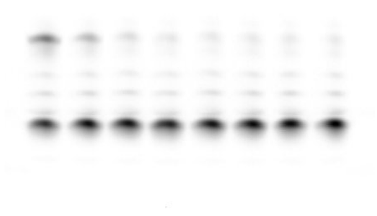

Figure S25: Uncropped gel image from the experimental replicate for Figure S4.

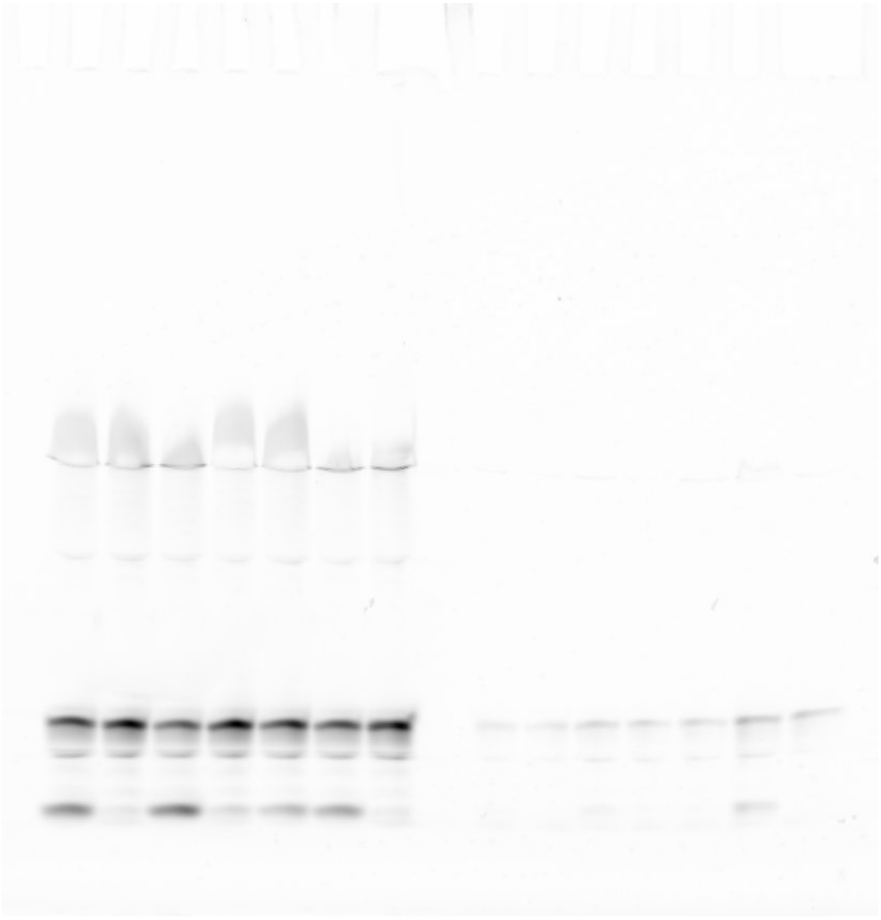

Figure S26: Uncropped gel image from Figure S5Ai.

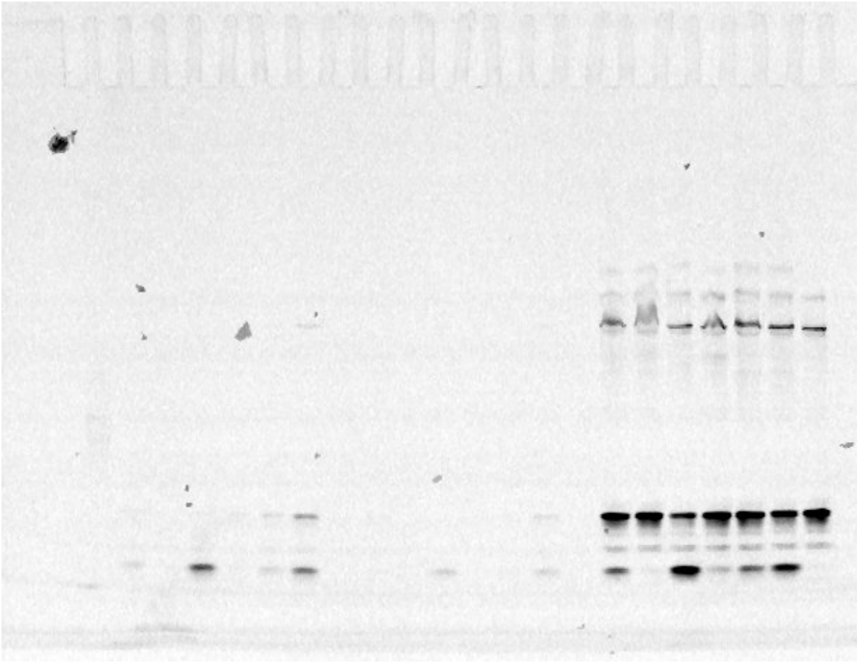

Figure S26: Uncropped gel image from Figure S5C.

10.0. Associated content and data availability

All raw data including uncropped gels have been provided in the data repository https://doi.org/10.17617/3.G6KSU2

11.0. Supplementary References

1. Saha, B., Chatterjee, A., Reja, A. & Das, D. Condensates of short peptides and ATP for the temporal regulation of cytochrome c activity. *Chem Commun (Camb)* **55**, 14194-14197 (2019).
2. Tabandeh, S. & Leon, L. Engineering Peptide-Based Polyelectrolyte Complexes with Increased Hydrophobicity. *Molecules* **24** (2019).
3. Waddell, W.J. A simple ultraviolet spectrophotometric method for the determination of protein. *The Journal of laboratory and clinical medicine* **48**, 311-314 (1956).
4. Spruijt, E., Westphal, A.H., Borst, J.W., Cohen Stuart, M.A. & van der Gucht, J. Binodal Compositions of Polyelectrolyte Complexes. *Macromolecules* **43**, 6476-6484 (2010).
5. Li, L. et al. Phase Behavior and Salt Partitioning in Polyelectrolyte Complex Coacervates. *Macromolecules* **51**, 2988-2995 (2018).
6. McCall, P.M. et al. Label-free composition determination for biomolecular condensates with an arbritarily large number of components. *bioRxiv* (2023).
7. Slaby, T. et al. Off-axis setup taking full advantage of incoherent illumination in coherence-controlled holographic microscope. *Optics Express* **21(12)**, 14747-14762 (2013).
8. Mittasch, M. et al. Non-invasive perturbations of intracellular flow reveal physical principles of cell organization. *Nat Cell Biol* **20(3)**, 344-+ (2018).
9. Voss, N.R. & Gerstein, M. Calculation of standard atomic volumes for RNA and comparison with proteins: RNA is packed more tightly. *Journal of Molecular Biology* **346(2)**, 477-492 (2005).

10. Zhao, H., Brown, P.H. & Schuck, P. On the distribution of protein refractive index increments. *Biophys J* **100**, 2309-2317 (2011).
11. Soumpasis, D.M. Theoretical-Analysis of Fluorescence Photobleaching Recovery Experiments. *Biophysical Journal* **41(1)**, 95-97 (1983).
12. Boeynaems, S., De Decker, M., Tompa, P. & Van Den Bosch, L. Arginine-rich peptides can actively mediate liquid-liquid phase separation. *Bio-protocol* **7**, e2525-e2525 (2017).
13. Hendry, P., McCall, M.J., Santiago, F.S. & Jennings, P.A. In vitro activity of minimised hammerhead ribozymes. *Nucleic Acids Res* **23**, 3922-3927 (1995).
14. Canny, M.D. et al. Fast Cleavage Kinetics of a Natural Hammerhead Ribozyme. *Journal of American Chemical Society* **2004**, 10848-10849 (2004).
15. Atkins, P., de Paula, J. & Keeler, J. Physical Chemistry, Edn. 11. (Oxford University Press, 2018).
16. Cuervo, A. et al. Direct measurement of the dielectric polarization properties of DNA. *Proc Natl Acad Sci U S A* **111**, E3624-E3630 (2014).
17. Malmberg, C.G. & Maryott, A.A. Dielectric Constant of Water from 0° to 100°C. *Journal of Research of the National Bureau of Standards* **56** (1956).
18. Muthukumar, M. 50th Anniversary Perspective: A Perspective on Polyelectrolyte Solutions. *Macromolecules* **50**, 9528-9560 (2017).
19. de Alcântara Pessôa Filho, P. & Maurer, G. An extension of the Pitzer equation for the excess Gibbs energy of aqueous electrolyte systems to aqueous polyelectrolyte solutions. *Fluid Phase Equilibria* **269**, 25-35 (2008).
20. Manning, G.S. Limiting laws and counterion condensation in polyelectrolyte solutions: IV. The approach to the limit and the extraordinary stability of the charge fraction. *Biophysical Journal* **7** (1977).
21. G.M, A. Error propagation by the Monte Carlo method in geochemical calculation. *Geochimica et cosmochimica acta* **40**, 1533-1538 (1976).
22. Hanke, F., Serr, A., Kreuzer, H.J. & Netz, R.R. Stretching single polypeptides: The effect of rotational constraints in the backbone. *Europhysics Letters* **92**, 53001 (2010).
23. Fitch, C.A., Platzer, G., Okon, M., Garcia-Moreno, B.E. & McIntosh, L.P. Arginine: Its pKa value revisited. *Protein Science* **24**, 752-761 (2015).
